# SPT: Single Pedestrian Tracking Framework with Re-Identification-Based Learning Using the Siamese Model

**DOI:** 10.3390/s23104906

**Published:** 2023-05-19

**Authors:** Sumaira Manzoor, Ye-Chan An, Gun-Gyo In, Yueyuan Zhang, Sangmin Kim, Tae-Yong Kuc

**Affiliations:** 1 Creative Algorithms and Sensor Evolution Laboratory, Suwon 16419, Republic of Korea; 2 Department of Electrical and Computer Engineering, College of Information and Communication Engineering, Sungkyunkwan University, Suwon 16419, Republic of Korea

**Keywords:** metric learning, person re-identification, Siamese network, single object tracking, convolutional neural network, YOLO, deep learning

## Abstract

Pedestrian tracking is a challenging task in the area of visual object tracking research and it is a vital component of various vision-based applications such as surveillance systems, human-following robots, and autonomous vehicles. In this paper, we proposed a single pedestrian tracking (SPT) framework for identifying each instance of a person across all video frames through a tracking-by-detection paradigm that combines deep learning and metric learning-based approaches. The SPT framework comprises three main modules: detection, re-identification, and tracking. Our contribution is a significant improvement in the results by designing two compact metric learning-based models using Siamese architecture in the pedestrian re-identification module and combining one of the most robust re-identification models for data associated with the pedestrian detector in the tracking module. We carried out several analyses to evaluate the performance of our SPT framework for single pedestrian tracking in the videos. The results of the re-identification module validate that our two proposed re-identification models surpass existing state-of-the-art models with increased accuracies of 79.2% and 83.9% on the large dataset and 92% and 96% on the small dataset. Moreover, the proposed SPT tracker, along with six state-of-the-art (SOTA) tracking models, has been tested on various indoor and outdoor video sequences. A qualitative analysis considering six major environmental factors verifies the effectiveness of our SPT tracker under illumination changes, appearance variations due to pose changes, changes in target position, and partial occlusions. In addition, quantitative analysis based on experimental results also demonstrates that our proposed SPT tracker outperforms the GOTURN, CSRT, KCF, and SiamFC trackers with a success rate of 79.7% while beating the DiamSiamRPN, SiamFC, CSRT, GOTURN, and SiamMask trackers with an average of 18 tracking frames per second.

## 1. Introduction

Visual object tracking (VOT) involves detecting and tracking the target object in video frames. It has received considerable attention due to its real-world applications in surveillance [1], traffic monitoring [2], robotics [3], activity recognition [4], video analysis for understanding human motion [5], medical diagnosis systems [6,7], and unmanned vehicles [8]. It refers to the trajectory estimation of the target object for precisely locating its position in subsequent video sequences. In recent years, researchers have made great efforts and provided many state-of-the-art (SOTA) trackers [9] to overcome different challenging factors, such as illumination variation, occlusion, scale change, deformation, and fast motion. However, despite significant progress, object trackers have not yet achieved ideal performance, and VOT remains an open challenge [10].

The development of VOT methods is divided into three stages [11]. The first stage started around the year 2000 with a focus on classification algorithms and machine learning-based target tracking [12,13]. However, despite their minimum computational requirements and fast speed, their accuracy and robustness were very low. The second stage of development was between 2010 and 2016, in which attention was paid to correlation filter-based trackers [14] that demonstrated good speed [15]. The third stage of VOT development, from 2016 to the present, has introduced deep learning-based Siamese models that are continuously improving to provide robust and more accurate trackers [16].

The Siamese network [17] is a y-shaped deep learning-based network that combines two parallel CNN models as core branches of its architecture where one branch takes a template patch (query or target image) as a region of interest (ROI) while the second branch takes candidate patches of the search region. It considers the tracking as a similarity learning problem [18] in which the template patch is extracted from a video frame as a target object, and it is matched with candidate patches in the current frame [19]. Metric-based similarity learning is used for model training. Recent works [20,21,22] have made seminal contributions with impressive performance using Siamese models, which address visual tracking as a universal similarity learning problem using an offline trained Siamese network [21].

VOT methods are classified into two categories known as single-object tracking (SOT) [23,24] and multi-object tracking (MOT) [25,26] models, which are trained online or offline.

In our work, we consider the problem of offline single-object tracking (SOT) in videos, where the object refers to the target pedestrian. The SOT process starts by specifying the initial position of the target with a bounding box in the first frame of the video sequence [27]. We use a convolutional Siamese network (CSN)-based offline SOT tracker that is trained on a large number of image pairs to learn the matching function during the training process, and then its performance is evaluated online on the videos. The main aim of using the Siamese network is to overcome the limitations of traditional deep learning-based CNN models. In our framework, we use the YOLO (you only look once) model for pedestrian detection and combine it with our Siamese-based re-identification model for performing detection-based tracking of the target person.

In this article, we make the following contributions.

1. We present an offline single object tracking framework for pedestrian tracking, which contains three main modules:
  (a) Pedestrian detection module.
  (b) Pedestrian re-identification module.
  (c) Tracking module that combines both detection and re-identification modules.
2. We propose two models for the pedestrian re-identification module:
  (a) MNet-Siam using transfer learning.
  (b) DCNet-Siam from scratch.
3. We evaluated both re-identification models on two datasets:
  (a) Large dataset: CUHK03.
  (b) Small dataset (our own collected dataset).
4. We performed a comparison of re-identification models with existing models.
  (a) We obtained improved accuracy.
5. We enhanced the architecture of the Siamese network by introducing the custom lambda block (CLB) in DCNet-Siam, which encourages the network to remove the tight dependency on the feature and custom head block (CHB) in MNet-Siam to improve its accuracy.
6. We combined the advantages of the deep learning-based one-stage detector and the metric learning-based Siamese re-identification model for data association in tracking module.
7. We performed qualitative and quantitative analyses with SOTA trackers for performance evaluation.
8. We performed practical experiments on both indoor and outdoor video datasets for single pedestrian tracking.
  (a) We obtained a higher success rate and tracking frames per second.

The rest of the article is divided as follows: Section 2 presents the literature review related to object detection, person re-identification, and single object tracking. Section 3 provides a detailed description of our single pedestrian tracking framework with its three modules and loss function. Section 4 reports the ablation study. Section 5 presents the results of each module and discusses the performance using different evaluation metrics. Finally, we conclude our work with an outline of future research direction in Section 6.

## 2. Literature Review

Our literature review section is divided into three subsections that are marginal to the topic of our work.

In the subsections of Section 2.1 and Section 2.2, we discuss the literature related to pedestrian detection and re-identification approaches; in Section 2.3, we present the studies related to the single object tracking models.

### 2.1. Pedestrian Detection

A considerable amount of research on object detection has been conducted using handcrafted [28,29,30,31,32], CNN-based [33,34,35,36], and hybrid [37,38,39,40] models. In handcrafted approaches, extracting multi-channel information is a challenging task. Zhang et al. [41] addressed this problem by developing a local, statistical multi-channel descriptor in which both color and gradient data were collected from each image patch to detect the articulations of the human body. Doll’ar et al. [42] developed several fast cascade structures for learning pedestrian features more efficiently and introduced the crosstalk cascade to overcome the computational costs. Deep learning methods based on CNN models have achieved significant success in pedestrian detection. In this direction, scale-aware pedestrian detection is a major challenge. Zhu et al. [43] presented the scale-adaptive deconvolutional regression (SADR) network to handle the problem of detecting pedestrians of different sizes; they incorporated multi-layer outputs to improve the detection performance. Liu et al. [44] introduced a scale-aware multi-resolution (SAM) method for selecting multi-resolution convolutional features according to pedestrian sizes and improved the performance by enhancing its model as SAM+ with feature channels. Yun et al. [45] designed a part-level CNN and performed pedestrian detection using salient features in the detection network to minimize the problem of false positives while using bounding box alignment in the alignment network to handle the proposal shift problem. In hybrid methods both handcrafted and CNN-based approaches are combined. Yang et al. [46] proposed a unified framework that combines lightweight filter channel features with a CNN model. This framework extracts features using pre-trained CNN models and feeds them to a boosting forest model for richer feature representation and lower computational cost compared to end-to-end CNN models.

### 2.2. Pedestrian Re-Identification

Pedestrian re-identification using metric learning based on classical and deep learning approaches has attracted the attention of researchers. In classical metric learning (CML) [47,48,49,50,51,52,53,54], certain rules are used to define the objective function. Syed et al. [55] proposed an adaptively weighted multi-kernel approach to improving the performance of person re-identification. They used Fisher discriminant analysis to enhance the robustness of classes. The approach first extracted color and texture features from image pairs and then mapped the extracted features. However, feature fusion-based metric learning may not accurately express the sample similarity and differences. To address this issue, Qi et al. [56] introduced person re-identification based on kernel learning with multi-feature subspaces. They achieved the distance metric and similarity functions by kernel learning and identified individuals by comparing the sum of the similarity of different feature subspaces. In deep metric learning (DML), the features are extracted using deep networks while the loss function is constructed to obtain the optimal parameters for feature extraction and reliable classification. Feng et al. [57] proposed a view-specific network model to overcome the challenge of intra-class variations. The model utilizes the cross-view Euclidean constraint to decrease the feature margin between different views and extends the cross-view center loss to provide a view-specific version. Varior et al. [58] presented a Siamese network and trained the model using contrastive loss by introducing a gating function for selectively emphasizing the common local patterns. Song et al. [59] used a binary segmentation mask for generating synthetic RGB-mask pairs as input and learned separate features of body and background region features by the mask-guided contrastive attention model for the person re-identification task. Chen et al. [60] boosted class-level contrastive ReID methods by leveraging the inter-instance pairwise similarity score using pairwise similarity ranking as one-hot pseudo labels for intra-class variance reduction and soft pseudo labels for the enhancement of different views to make the models more robust.

### 2.3. Single Object Trackers

Single object trackers are also known as visual object trackers, which are mostly categorized as correlation filter-based and deep learning-based trackers. In a correlation filter-based tracker (CFT), Bolme et al. [15] introduced the minimum output sum of the squared error (MOSSE) filter in which the target appearance is modeled by correlation filters while single object tracking is performed via convolution. Henriques et al. [61] proposed a kernelized correlation filter (KCF) to handle thousands of data patches. They explored the properties of circulant matrices and the tools of kernel tricks to reduce the computation load. Li et al. [62] addressed the fixed template size problem in the KCF tracker by introducing the scale adaptive scheme and improved overall performance by integrating HoG and color features. CFT highly depends on the spatial layout of the target, which makes CFT sensitive to deformation. Bertinetto et al. [63] tackled this issue by proposing a staple tracker in which color cues were combined with template models. Galoogahi et al. [64] proposed a tracker to implicitly learn correlation filters by exploiting all possible patches over an embedded dense sampling strategy. It further improves performance by reducing the circular boundary effects and taking the computational advantages of the frequency domain. Discriminative correlation filter (DCF)-based approaches [15,61,62] rely on the periodic assumptions of training samples that generate unwanted boundary effects that degrade the quality of the tracking model due to inaccurate representations of image content. Danelljan et al. [65] proposed spatially regularized discriminative correlation filters (SRDCF) to overcome this problem and introduced a spatial regularization component in the learning to improve the discriminative appearance model. Alan et al. [66] improved DCF trackers by introducing channel and spatial reliability concepts. They provided efficient integration in the filter update and the tracking process through the learning algorithm. Spatial reliability maps are used to provide the ability to track non-rectangular objects and channel reliability weights are used to reduce the noise of the weight-averaged filter response. In recent years, the majority of research has been focused on deep learning-based (DL-based) networks, and most researchers apply DL-based models to improve the object tracking results. In this direction, Reference [67] proposed a multi-domain learning network (MDNet) by training a CNN model offline to obtain the target representation and fine-tuning the model online after concatenating a domain-specific layer with the pre-trained CNN for tracking the target object. A seminal study in this area is based on the fully convolutional (FC) Siamese models. Held et al. [68] introduced generic object tracking using regression networks for efficient object tracking and used frozen weights of the networks to track the target object without any need to fine-tune the model online. David et al. [68] introduced GOTURN, a CNN-based tracker that is faster due to its offline training and does not require online fine-tuning. It learns the generic relationship between motion and appearance of the object to track novel objects that do not appear in the training set using a sequence of convolutional layers. Bertinetto et al. [21] proposed SiamFC, which is considered a cornerstone of Siamese tracking and emphasizes the fully convolutional architecture for similarity learning. It uses a larger search region instead of a search image of the same size as the exemplar image and calculates the similarity on a dense grid. Hu et al. [69] combined object tracking with segmentation using a multi-task learning approach to perform three main tasks, i.e., bbox regression, similarity learning, and class-agnostic binary segmentation. Its architecture was based on the modified ResNet-50 [70] with an adjustment layer. It refines the object information by focusing on the class-agnostic binary segmentation and creates a multi-channel response map by depth-wise cross-correlation [71]. Zheng [72] presented a distractor-aware Siamese model based on a region proposal network (DaSiamRPN) to improve the robustness of Siamese trackers by distinguishing the foreground from the semantic background. They introduced a local-to-global search strategy to handle occlusion and out-of-view challenges and re-detect the target.

## 3. Proposed Framework

In this paper, we propose a convolutional Siamese-based pedestrian re-identification model for locating the target person from the candidate patches through the matching function. The candidate patches are extracted using the YOLOv8 single-stage detector. The matching function of the re-identification model is learned offline on training data in terms of image pairs, and it is employed with the template branch and search branch. Both CNN branches share the same weights to learn the similarity between the target and the candidate patches.

Our single pedestrian tracker (SPT) consists of three main modules. In this section, we explain the overall architecture and workflow of our framework in detail as follows:

1. Detection module;
2. Pedestrian re-identification module;
3. Tracking module.

### 3.1. Detection Module

We employ YOLOv8 [73] for pedestrian detection, which is a recently launched state-of-the-art object detection model developed by Ultralytics and was launched on 10 January 2023. YOLOv8 uses a one-stage object detection approach, in which a single neural network is applied to the entire image. Its performance is better compared to similar models and it includes numerous architectural improvements. Currently, it is under active development and does not have a published paper at the time of writing our article. Therefore, we lack direct insight into the model architecture.

It is an anchor-free model that predicts the object’s center directly instead of using anchor boxes. This reduces the number of box predictions and speeds up Non-Maximum Suppression (NMS). YOLOv8 replaces C2f [74] (which concatenates all outputs from the bottleneck) with C3 [75], which only uses the output of the last bottleneck.

The Bottleneck module in YOLOv8 is the same as in YOLOv5 [76], but the size of the first convolutional kernel is changed from 1 × 1 to 3 × 3. The Neck module concatenates the features directly, reducing the overall size of tensors and parameter count. YOLOv8 uses a decoupled head and deletes the objectness branch [77]. During training online, mosaic augmentation is used, in which four images are stitched together to learn the objects at different locations with or without partial occlusion.

YOLOv8 has five versions, ranging from YOLOv8n (the smallest model) to YOLOv8x (the largest model). YOLOv8n provides the highest FPS due to its small size; however, it scores the lowest among all YOLOv8 variants with a 37.3% mAP. While YOLOv8x offers the highest 53.9% mAP score, its FPS is low due to the extra-large size. Therefore, we have selected YOLOv8m, the medium model, because it lies between YOLOv8n, YOLOv8s, YOLOv8l, and YOLOv8x in terms of performance with a 50.2% mAP score [73].

### 3.2. Pedestrian Re-Identification Module

In the pedestrian re-identification module, our main contribution is that we propose two different network architectures. One network is based on a pre-trained model while the second model is trained from scratch.

#### 3.2.1. Model 1: From Scratch

In the following subsection, we will explain the architecture of our proposed model in two parts. The first part consists of its backbone, called the deep convolutional neural network (DCNet), while the second part explains its overall architecture, called DCNet-Siam.

##### DCNet: Backbone Architecture

**Figure 1:**
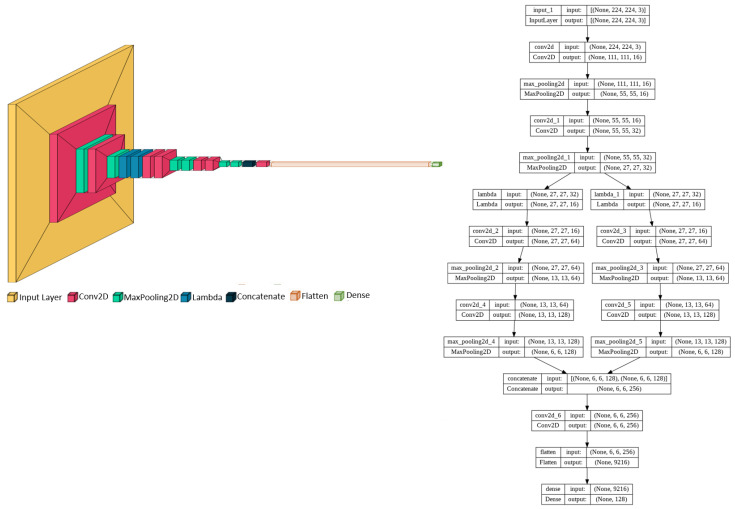
Model 1: DCNet backbone proposed from scratch.

The backbone architecture of the DCNet model contains six convolutional layers for the base network, as given in Figure 1. The first 2 layers of the DCNet CNN architecture are convolutional layers with 16 and 32 filters of size 3 × 3. These layers perform the convolution process, which is followed by normalization and pooling. Convolution is a mathematical operation shown in Equation (1), which is applied both horizontally and vertically to the input image *I* using kernel *F*.

(1) $$F*I\left(x,y\right)=\sum_{j=-N}^{N}\sum_{i=-N}^{N}F\left(i,j\right)I\left(x-i,y-j\right)$$

We used padding in each convolutional layer for two main reasons. First, the original image size shrinks at each step after multiple convolution operations in each layer, which we do not want. Second, overlap occurs when the kernel passes through the middle layers more times. Therefore, we used zero-padding as the “same” inside the convolutional layers, which adds extra pixels outside the image border to preserve its original size. The input image contains a lot of non-linear features such as transitions between colors, pixels, and borders. For this, we used ReLU with each convolutional (including a fully connected layer to handle naturally non-linear images) in our DCNet model.

The original image size shrinks after each convolutional operation. Therefore, after each convolutional layer, we used a batch normalization layer that focuses on standardizing the inputs from previous layers by maintaining the mean output close to 0 and the standard deviation close to 1. It speeds up the training by a large margin by dealing with the sequences of different scales. The last process in our model involves convolutional and normalization layers followed by the pooling process. The feature maps obtained from the convolutional layers are sensitive to the location in the input. Downsampling the feature maps is an approach to address this sensitivity by making the extracted feature maps more robust to changes in the feature position inside the image. This approach, known as “local translation invariance”, is applied using pooling layers in our model to downsample the detection of features in feature maps. Mean pooling and max pooling are two common methods for performing the pooling operation. Mean pooling returns the average of all values for each patch covered by the kernel size on the feature map, while max pooling calculates the maximum value from the portion of the feature maps retained by the kernel. Max pooling returns shapes with sharp edges, while mean pooling summarizes the shapes in neighborhood pixels. In our DCNet architecture, we used the max pooling operation by defining the maxpooling2D layer with a kernel size of 3 × 3 and a stride of 2, which downsamples each feature map by reducing its size to one-quarter with an overlapping factor of 2. However, the number of filters increases as we go deeper into the network, which generates correspondingly larger feature maps that consume more memory. Therefore, at our third layer, we slice the input feature map using a custom lambda layer, which splits the feature maps in half into two blocks of layers of the same size, removing the tight dependency between the layers’ feature maps. The fourth and fifth layers convolve each block with a 3 × 3 kernel, and normalization and pooling operations are performed on each block. The final output of the two blocks of layers is the input to the sixth layer, which concatenates them. We call this the custom lambda block (CLB). In the 7th layer, we perform a convolution with 256 filters of size 3 × 3 without a max pooling or normalization process. The concatenated layers of both blocks are flattened into one long vector at the eighth layer. The 9th and 10th layers are fully-connected layers with a 128-unit vector followed by dropout regularization to handle the over-fitting problem. Finally, we use an array of layers to construct the model that outputs the feature embeddings. Hence, the base network is also called an embedding network, and it serves as the feature extractor.

##### DCNet-Saim: Pedestrian Re-Identification Architecture

**Figure 2:**
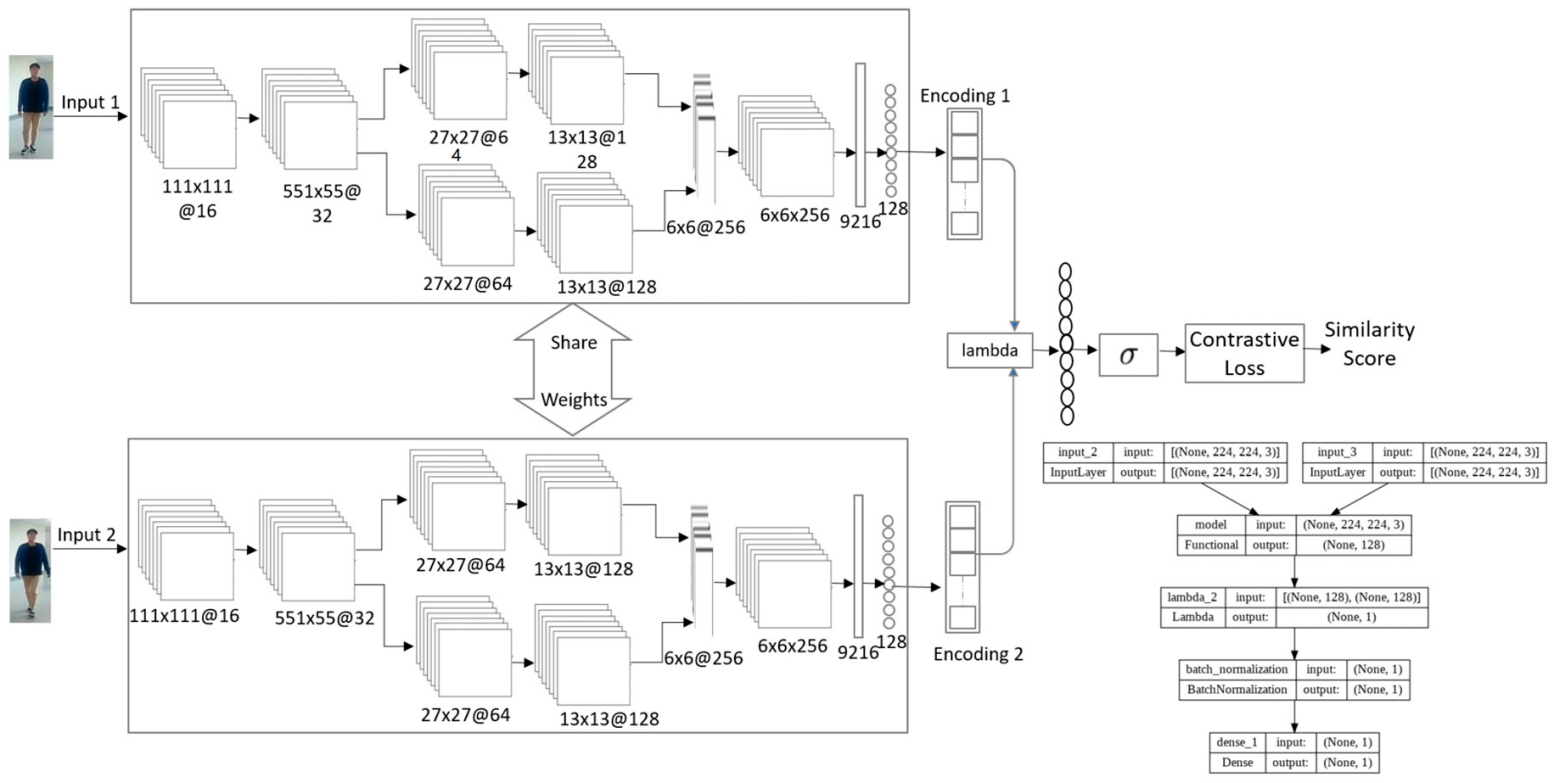
Model 1: The architecture of our proposed DCNet-Siam for pedestrian re-identification.

The complete architecture of our DCNet-Siam model consists of two DCNet subnetworks, as shown in Figure 2.

Overall, the architecture of our DCNet-Siam model consists of two embedding networks as the base models. Each embedding model contains a total of eleven layers; after two convolutional layers, we introduce one custom lambda block (CLB), one concatenation layer, one flattened layer, and one dense layer. The first convolutional layer uses 3 × 3@16 kernels, the second convolutional layer uses 3 × 3@32 kernels, the third and fourth convolutional layers use 3 × 3@64 kernels for both top and bottom groups, while the fifth and sixth convolutional layers use 3 × 3@128 kernels for the top and bottom branches. The seventh convolutional layer is added after concatenating the feature maps of the top and bottom layer branches. Max pooling is employed from the first to the sixth layer using 3 × 3 pooling and 2-pixel strides after batch normalization. However, the seventh layer is a convolutional layer without pooling and batch normalization. After the second convolutional layer, a custom lambda layer is used to split the feature map in half into 16 × 16. After the 7th convolutional layer, the multi-dimensional inputs are flattened into a 1-dimensional vector with 9216 neurons, which is then passed to the fully connected layer with dropout regularization. The final fully connected layer, also called the embedding layer, extracts a feature vector of 128. The input to the DCNet model is a 3-channel RGB image with 224 × 244@3 resolution, containing a total of 1,941,222 neurons.

The DCNet-Siam model takes two images as input pairs. Both images are passed to the DCNet embedding networks, which are twin models that share the same weights. Each embedding network outputs a 1D array with the desired size of the 128-dimensional feature vector. These feature vectors obtained from the embedding networks are passed to the Lambda layer, which calculates the distance between the two vectors. After that, we apply batch normalization to regularize the model. Then, we use a dense layer consisting of a single neuron with a sigmoid activation function, which outputs a value between 0 and 1. Values closer to zero indicate that the image inputs passed to the network as pairs are less similar, while values closer to one imply that the template image is more similar or likely to be in the search region. This completes the overall architecture of our DCNet-Siam model, which serves as the verification network.

#### 3.2.2. Model 2: Using Transfer Learning

In the following subsection, we explain the architecture of our transfer learning-based model in two parts. The first part consists of its backbone, called MobileNet (MNet), while the second part explains its overall architecture, called MNet-Siam.

##### MNet: Backbone Architecture

We used a transfer learning-based approach to construct the MNet model with the MobileNet-v2 [78] architecture. Transfer learning [79] allows for the reusing of the parameters and weights of pre-trained models for new problems. We selected MobileNet-v2 because it is suitable for running on mobile and edge devices, such as Jetson Xavier NX. Its architecture, consisting of 53 layers, contains fewer parameters, making it smaller in size compared to other pre-trained models [70,80,81]. With pre-trained mobileNet-v2 as the backbone for feature extraction, we take the weights from the “imagenet” dataset and do not retrain the backbone on the previous dataset. Instead, we freeze the base model by setting the trainable parameter to “False” and create a custom head block (CHB) on top of the last layer. For this block, we discard the last fully connected layer and replace it with the “globalaveragepooling” layer. Next, we add two dense layers, which are followed by the “batchnormalziation” and “dropout” regularization. Finally, we build the embedding network, which generates a 128-dimensional feature vector as an output of the MNet model.

##### MNet-Saim: Pedestrian Re-Identification Architecture

**Figure 3:**
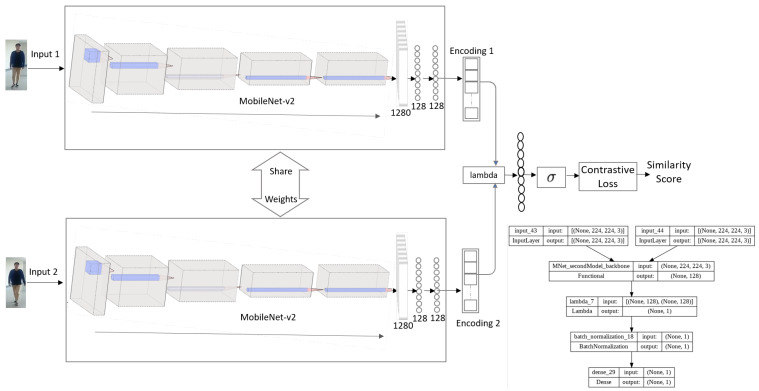
Model 2: The architecture of our proposed MNet-Siam for pedestrian re-identification.

The architecture of MNet-Siam, consisting of twin mobileNet-v2 models as the backbone networks, is shown in Figure 3.

Overall, the architecture of our MNet-Siam model consists of two MNet base models as embedding networks that take two input images as pairs and share the weights. Each embedding network outputs a 1D array with the desired size of a 128-dimensional feature vector, which is passed to the next layer for finding the distance between them. This layer is followed by batch normalization and a dense layer with a single unit for binary classification using the sigmoid activation function. The less similar pairs are represented with an output closer to zero, while the pairs with higher similarity are predicted with a value closer to one. This completes the person re-identification/verification network of our transfer learning-based MNet-Siam architecture.

### 3.3. Tracking Module

This module combines both detection (Section 3.1) and re-identification (Section 3.2) models to build a practical application for single pedestrian tracking (SPT) in the videos.

**Figure 4:**
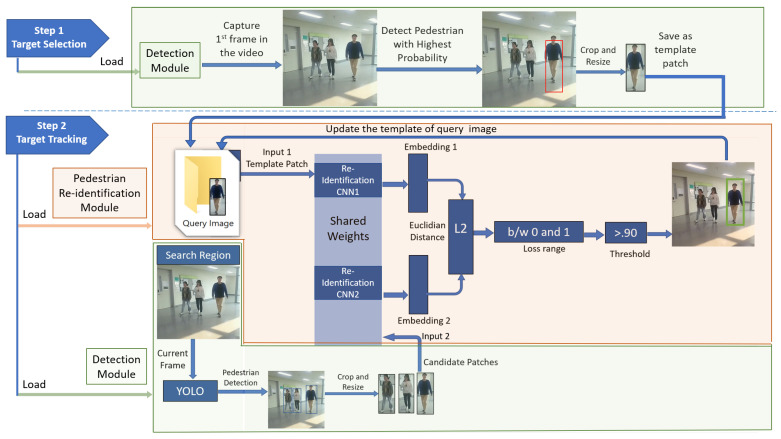
Runtime process of the proposed SPT framework based on detection and pedestrian re-identification models.

**Figure 5:**
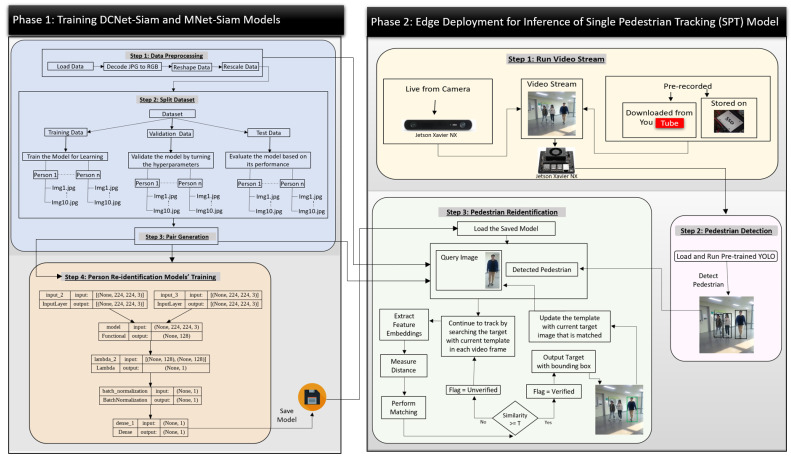
The overall workflow diagram of the SPT framework from training to deployment.

The overall architecture of our framework is shown in Figure 4 while the workflow of its complete process is presented in Figure 5.

Figure 4 shows that our framework uses a tracking-by-detection approach in which we combine YOLO-based detection with our proposed Siamese-based re-identification models. Pedestrian detection was performed using YOLOv8 [73] while pedestrian verification in each frame was performed using proposed re-identification models for single pedestrian tracking.

Figure 5 shows the workflow diagram of our SPT framework, which consists of two phases. The first phase is divided into two steps in which the first step performs data preparation, while the second step is training. Phase 2 represents the deployment workflow of our model on Jetson Xavier NX for single pedestrian tracking (SPT) in video streams to identify the target.

At runtime, we perform two steps, as illustrated in Figure 4. The first step is to select the target pedestrian while the second step is to perform the target re-identification in video frames for detection-based tracking.

Step 1: Target initialization as query image at runtime.

We used a pre-trained YOLOv8 [73] model to detect the target pedestrian in a single frame and track the first person we found with the largest probability. For this, our framework is called the detection module, which utilized YOLOv8 [73]. As shown in Figure 4, when we run our framework with a live camera stream, it captured the first frame in which three people were standing in front of the camera. In the detection module, we first ensured that at least one detection was made from the person class. After that, we found the index of the detection with the largest probability. Next, we used bounding boxes to obtain the coordinates of the detected pedestrian and cropped the region associated with the highest probability. Finally, we saved it as our query image that represented the target pedestrian in the first video frame.

Step 2: Target tracking based on detection and re-identification models.

The query image, extracted as a template patch in Step 1, is passed as input 1 to our pedestrian re-identification model, while the current frame is used as a search region that is given to the detection module. In this module, YOLOv8 [73] detects the pedestrian to extract the candidate patches, which are then passed to the re-identification model as input 2.

The re-identification module takes both the query image and candidate patches of pedestrians as input pairs, as shown in Figure 4. It extracts the feature embeddings and calculates the Euclidean distance to measure the similarity between the template and candidate patches. The patches are considered matched if the similarity between them is greater than the specified threshold, as shown in Figure 5. As a result, the module outputs a bounding box around the matching patch in the video frame and updates the template image with the matching image, showing the new position of the target pedestrian in the video frame. This process is repeated to re-identify the target with an updated template for tracking in the remaining video frames.

### 3.4. Loss Function

**Figure 6:**
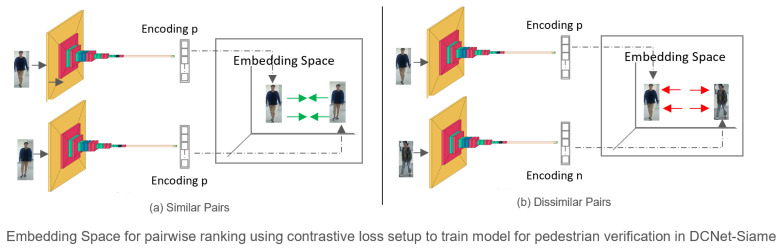
Embedding Space for pairwise ranking using contrastive loss.

The selection of the right loss function is important because it significantly impacts the model explanation, interpretability, and its ability to fit the training images. For solving the pedestrian re-identification problem, we selected the distance metric learning-based contrastive loss shown in Equation (2) due to its ability to operate on data points directly instead of comparing classification results. This loss function matches the predicted and target output values of the pairs to determine how well the Siamese network can model the training data for distinguishing between image pairs. Training the model with this loss function requires a pair of positive and negative images. The objective of the contrastive loss is to embed the encodings in the latent space as shown in Figure 6 with a small distance for positive pairs and a large distance for negative pairs.

(2) $$ContrastiveLoss=Y{\left(E\right)}^{2}+\left(1-Y\right)max{\left(m-E,0\right)}^{2}$$

*Y* is the ground truth label that can be either 0 or 1. It will be 0 when the input pairs are from the same class; otherwise, Y’s value is 1.

${\left(E\right)}^{2}$ is the square of the Euclidean distance (or squared Euclidean distance) between two pairs with respect to their weights. The reason for using the squared Euclidean distance metric is that it allows for faster clustering compared to the regular Euclidean distance. It uses the same equation as the Euclidean distance metric shown in Equation (2); however, it does not take the square root.

(3) $$EuclideanDistanc=\sqrt{{}^{(x_{2}-x_{1})2}{+}^{(y_{2}-y_{1})2}}$$

*m* is the margin. Typically, its value is 1. It holds the constraint, which is when two input values are dissimilar. If it is set to 0, then the loss function would not be able to separate difficult encoding pairs because it will give them the same value.

0 indicates that the images belong to two different classes.

The max function takes the largest value of 0 and the margin, m, minus the squared distance.

If we look at the loss function in Equation (2), it is composed of two parts.

1. When the image pairs are similar, the label becomes zero (y = 0) and only the first part of the loss function is executed, while the right-hand side additive part is omitted. In this case, the loss becomes the distance of two encodings extracted from two similar images, and E is minimized using squared distance to ensure that the model is penalized if it gives a high distance for similar encodings.
2. When the image pairs are dissimilar, the label of Y becomes Y = 1, and only the second part of the loss function executes while the first left-hand side part is excluded. The loss function is based on the distance between dissimilar encodings, and E is maximized to the margin. The margin m ensures that the embeddings produced for a negative pair are distant enough. If the image pairs are well separated with the defined margin, then the error contribution will be zero. For example, suppose we choose 10 for the margin, and 17 and 1 as the predicted Euclidean distances between two encoding pairs (e1,e2) and (e3,e4).
  For encoding_pair_1 = $max{\left(10-17,0\right)}^{2}=max{\left(-7,0\right)}^{2}=0^{2}=0$, with no contribution to the error. For encoding_pair_2 = $max{\left(10-1,0\right)}^{2}=max{\left(9,0\right)}^{2}=9^{2}=81$, with high contribution to the error.

In the above example, the distance between e1 and e2 is 17 in encoding_pair_1, which is greater than margin 10. Therefore, encoding_pair_1 is well separated and does not contribute to the error.

However, the loss function needs to focus on separating the dissimilar encoding pairs with a smaller distance between them. In this case, the margin helps in optimization to embed the encoding pairs that do not have enough distance. In the above example, the distance between e2 and e3 in encoding_pair_2 is 1, which is very small. However, the loss function has high errors due to the use of margin, which helps to correctly separate them as different encodings.

To recap, if there is a large distance between encodings, they can be easily separable with zero error, but if the distance is small, it can be difficult to separate them. In this case, the margin plays a crucial role by increasing the contribution error to embed dissimilar encoding pairs correctly despite the small distance between them. Consequently, the loss is low when similar pairs are encoded to closer representations, while dissimilar pairs are encoded to farther representations.

## 4. Ablation Study

The main components of our proposed models are custom blocks of layers that enhance the architecture of Siam models and play an important role in improving accuracy. An ablation study was conducted on the CUHK03 [82] dataset to evaluate the extent of the contribution of each component as shown in Table 1.

For DCNet-Siam, we conducted three experiments to verify the effectiveness of our custom lambda block (CLB): (a) without CLB, (b) using CLB without the feature map split (FMS), and (c) using CLB with the feature map split, by half, into two groups. The results of the ablation study reported in Table 1 show that the proposed CLB with FMS achieves a 7.5% increase in accuracy. Using CLB after removing FMS results in an approximate 2.8% drop in performance. Removing both CLB and FMS further reduces the rank-1 accuracy to 4.7%.

For MNet-Siam, to verify the effectiveness of the custom head block (CHB), we performed two experiments: (d) without CHB and (e) with CHB in the backbone network, as shown in Table 1. Our ablation study shows that introducing the custom head block (CHB) leads to a 3.6% increase in the results of the rank-1 accuracy compared to the architecture without CHB in the base network.

## 5. Results

In this section, we illustrate the experimental results in detail by comparing our models with state-of-the-art (SOTA) models using different evaluation criteria. We divided the results into two subsections. In the first subsection, Section 5.1, we discuss the results of the person re-identification module. In the second subsection, Section 5.2, we explain the experimental results with a detailed discussion and analysis of the video dataset for the tracking module.

### 5.1. Module 2: Pedestrian Re-Identification

Our proposed pedestrian re-identification models, MNet-Siam and DCNet-Siam, were trained and tested on two datasets: CUHK03 [82] as a large dataset and our own collected data as a small dataset. For the performance evaluation of our re-identification module, we considered the computational cost and accuracy as evaluation metrics.

- Computational cost: Models with higher performance require more computing power and time. The total number of parameters of the model is a good indicator of the computational cost. A large number of parameters shows that the model requires more calculation time to train the network.
- Accuracy: The top-1 accuracy is a good estimator of performance in the model selection. It measures the performance in terms of the time taken by a transfer learning-based model to make a correct top-class prediction.

#### 5.1.1. Results on Large Dataset

**Figure 7:**
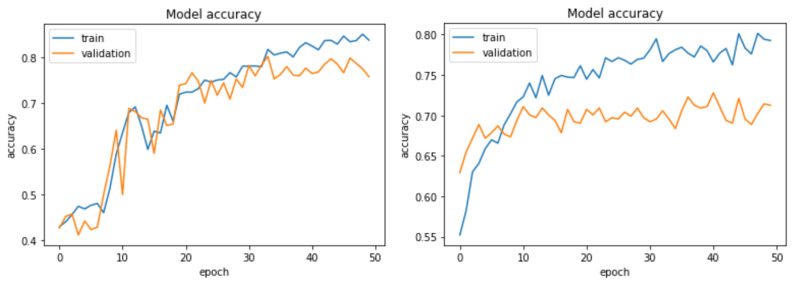
Accuracy curve of our proposed pedestrian re-identification models on the CUKH03 dataset.

**Figure 8:**
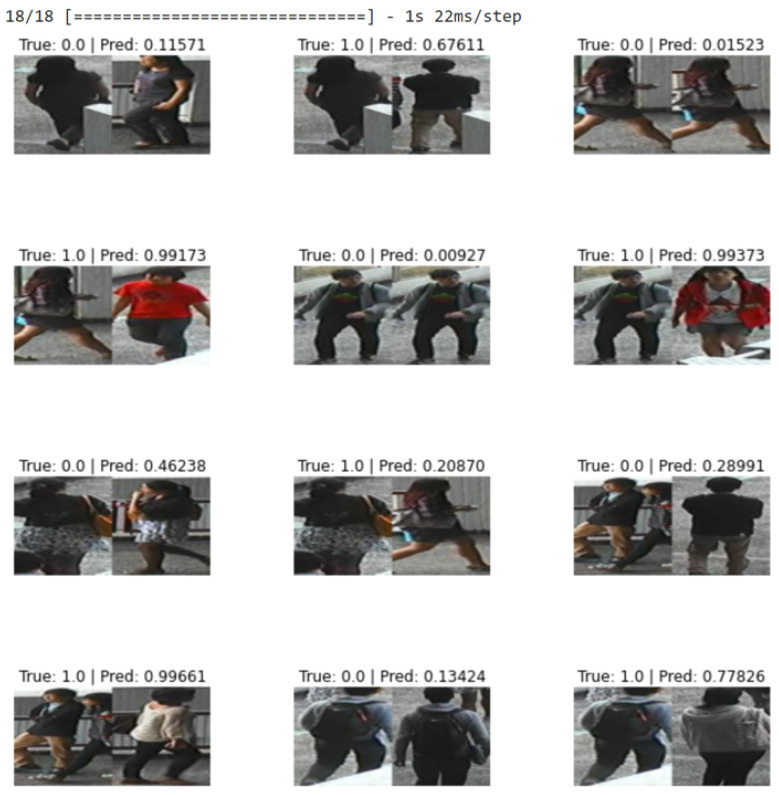
Results of our proposed pedestrian re-identification models on the CUKH03 test dataset.

For the large dataset, experiments were conducted using CUKH03 [82] dataset. Figure 7 shows that our DCNet-Siamese obtained 83% accuracy while our MNet-Siam achieved 79% accuracy. The results of the test data are shown in Figure 8.

**Figure 9:**
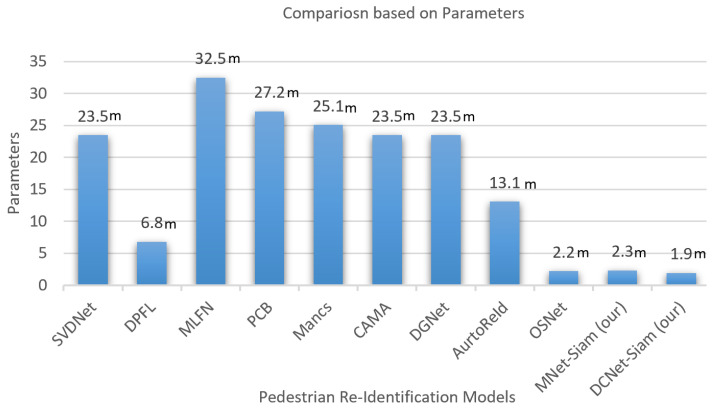
The comparison with existing pedestrian re-identification models based on the number of parameters shows that our proposed models are lightweight.

Table 2 illustrates the comparison of our models with SOTA methods on the CUHK03 dataset. Figure 8 shows the results of our proposed models. Figure 9 demonstrates that DCNet-Siam has the lowest parameters and depth compared to MNet-Siam. Moreover, our Siamese networks achieve a much smaller model size compared to other person re-ID models, which are mostly based on pre-trained networks, such as ResNet50 [83,84,85,86] having parameters from 23 million to 27 million and InceptionNet [87] having parameters more than 6.8 million. In contrast, our TL-based MNet-Siam model has 2.3 million parameters, while our DCNet-Siam model proposed from scratch has only 1.94 million parameters.

**Figure 10:**
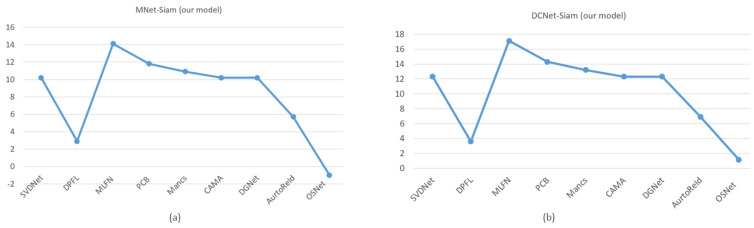
How small or large are the MNet-Siam (**a**) and DCNet-Siam (**b**) models compared to other models.

Figure 10 shows that our proposed MNet-Siam with the transfer learning-based approach is a lightweight model compared to the rest, except OSNet [88] and our DCNet-Siam. It is 14.1×, 11.8×, 10.9×, 5.7×, and 2.6× smaller than MLFN [89], PCB [83], Mancs [84], AutoReId [90], and DPFL [87], and 10.2× smaller than SVDNet [91], CAMA [85], and DGNet [86]. Notably, Figure 10b shows that the proposed DCNet-Siam from scratch with a custom backbone architecture is the lightest-weight model among all. It can be seen that DCNet-Siam is 17.1×, 14.3×, and 13.2× smaller than MLFN [89], PCB [83], and Mancs [84], and 12.3× smaller than SVDNet [91], CAMA [85], and DGNet [86]. Similarly, it is also lighter than the smaller models, including AutoReId [90], DPFL [87], OSNet [88], and our MNet-Siam by 6.9×, 3.6×, 1.1× and 1.2×, respectively.

**Figure 11:**
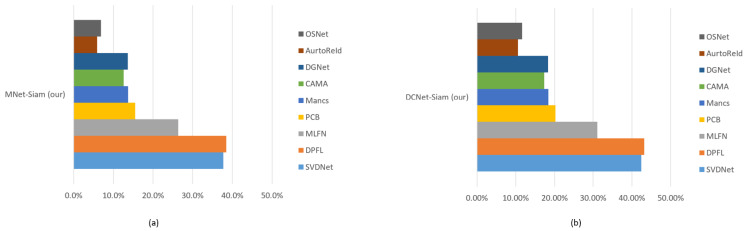
Increase or decrease in accuracy of MNet-Siam (**a**) and DCNet-Siam (**b**) compared to other models.

Table 2 shows that the proposed TL-based MNet-Siam obtains 79.2% accuracy, which is higher compared to all re-identification models except for DCNet-Siam. Figure 11a illustrates that its accuracy for the person re-identification task is 37.7%, 38.5%, 26.4%, 15.5%, 13.7%, 12.6%, 13.6%, 5.9%, and 6.9% higher than SVDNet [91], DPFL [87], MLFN [89], PCB [83], Mancs [84], CAMA [85], DGNet [86], AutoReId [90], and OSNet [88] respectively.

Table 2 also demonstrates that our proposed S-based DCNet-Siam achieves the best overall performance with the highest accuracy of 83.9% on the large pedestrian re-id dataset and it surpasses all other models with a clear margin. It was trained from scratch by proposing a new pedestrian re-identification model, which has accuracy increases of 10.6 and 11.6 compared to AutoReID [90] and OSNet [88] models that are also trained from scratch, as shown in Figure 11b. In addition to that, it can be seen that the performance accuracy of DCNet-Siam is also higher than transfer learning-based models. Figure 11b shows that compared to SVDNet [91], DPFL [87], MLFN [89], PCB [83], Mancs [84], CAMA [85], and DGNet [86], it improved the accuracy by 42.4%, 43.2%, 31.1%, 20.2%, 18.4%, 17.3%, and 18.3%, respectively.

**Figure 12:**
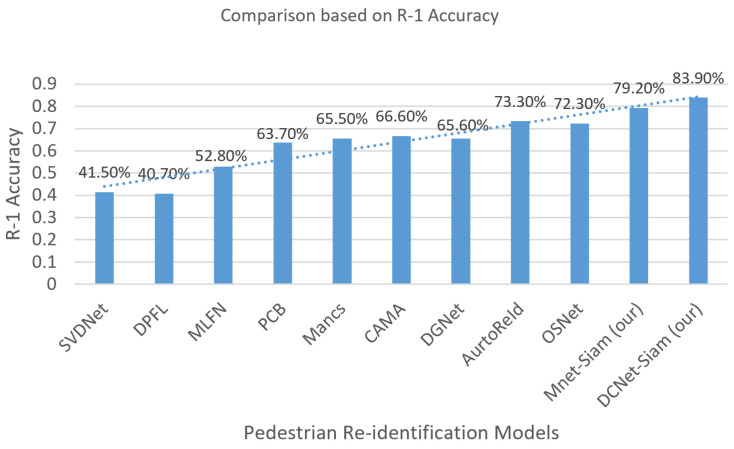
Comparison between existing pedestrian re-identification models based on the R1 accuracy demonstrate that our proposed models have the highest accuracy.

To summarize, we compared our proposed models with nine SOTA models in terms of their size (lightweight) and accuracy. Our MNet-Siam is lighter than eight models, while DCNet-Siam is the lightest among all models, including MNet-Siam, as shown in Table 2. Although most top-performing transfer learning-based models rely on the ResNet50 backbone with a large number of parameters such as [85,86] having more than 23.5 million parameters, our transfer learning-based MNet-Siam with 2.3 million parameters is more lightweight. Our DCNet-Siam is even lighter than our MNet-Siam model, with only 1.9 million parameters. Large models contain more parameters and, therefore, are more prone to overfitting the training data. However, the results demonstrate that our proposed MNet-Siam and DCNet-Siam models achieved improvements in accuracy with fewer parameters, as shown in Figure 10 and Figure 12. They beat other models trained from scratch [88,90] and the models trained using transfer learning methods [83,84,85,86,87,89,91], as shown in Table 2. These results verify the effectiveness of our proposed models for the person re-identification task.

**Table 2:** Results on the CUHK03 dataset compared with other models for pedestrian re-identification. It is noteworthy that the proposed MNet-Siam pedestrian re-identification model surpasses most published models except for OSNet, while our proposed DCNet-Siam beats all models in terms of accuracy and parameters, making it a light and compact model.

	#	Siamese Models	Base Models	Parameters	R1 Accuracy on CUHK03
	1	SVDNet [91]	ResNet	>23.5	41.5%
	2	DPFL [87]	InceptionNet	>6.8	40.7%
	3	MLFN [89]	ResNetXt	32.5	52.8%
	4	PCB [83]	ResNet	27.2	63.7%
	5	Mancs [84]	ResNet	>25.1	65.5%
	6	CAMA [85]	ResNet	>23.5	66.6%
	7	DGNet [86]	ResNet	>23.5	65.6%
	8	Auto-ReID [90]	Auto	13.1	73.3%
	9	OSNet [88]	OSNet	2.2	72.3%
Our Models	10	MNet-Siam	MobileNet-v2	2.3	79.2%
	11	DCNet-Siam	DCNet	1.9	83.9%

#### 5.1.2. Results on Our Small Dataset

**Figure 13:**
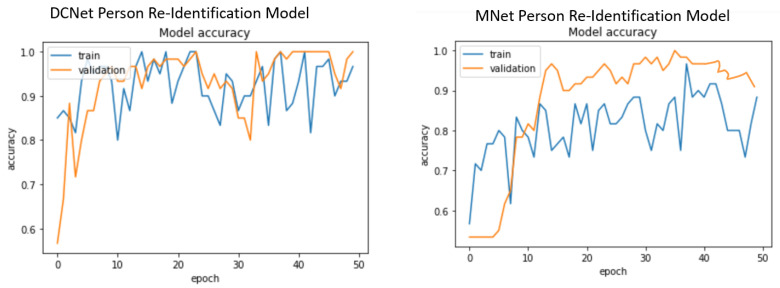
Accuracy curve of our proposed DCNet and MNet pedestrian re-identification models on the small dataset.

**Figure 14:**
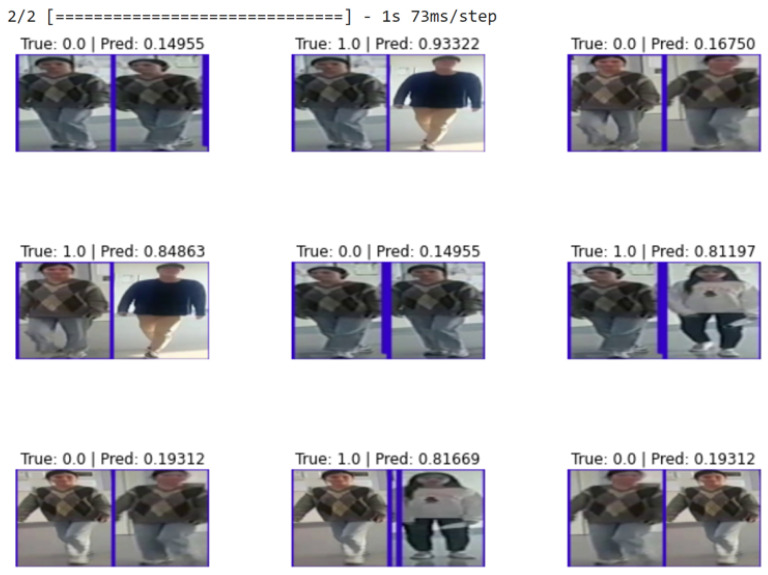
Results of our proposed pedestrian re-identification models on the small test dataset.

Obtaining good results on a small dataset for the pedestrian re-identification task is more challenging because they have fewer training images compared to a large dataset. However, in contrast to deep CNN models, the purpose of metric learning-based Siamese models is to perform prediction with few training examples. To evaluate our re-identification models on a small dataset, we collected our own pedestrian dataset of walking people. Images were extracted by recording videos from different viewpoints (front, back, left, and right sides). For the indoor environment, our small dataset consists of 30 training images of three identities walking in the corridor while there are 10 testing and 10 validation images. Figure 13 shows the accuracy of our re-identification models trained on a small dataset while the results on the test data are shown in Figure 14.

To summarize, the test accuracy of our MNet person re-identification model is 92%, while our DCNet person re-identification model is 96%, which is 4% higher than the MNet model on the small dataset, as shown in Figure 13.

### 5.2. Module 3: Pedestrian Tracking

For performing the experiments, we used DCNet-Siam instead of MNet-Siam because the experimental results in Section 5.1 demonstrate that the DCNet is more efficient in terms of accuracy and computational costs. Therefore, we combined YOLOv8 [73] for pedestrian detection and the DCNet-Siam for data association in the tracking module.

To evaluate the performance of our tracking module, we conducted both qualitative and qualitative analyses by running our tracker on six video streams in both indoor and outdoor environments and compared our tracker with SOTA SiamFC [21], CSRT [66], DiaSiamRPN [72], GOTURN [68], KCF [61], and SiamMask [69] trackers.

#### 5.2.1. Qualitative Analysis

We performed the qualitative analysis based on the visual representation of the results by considering six major challenging factors of the real-world environment, which were observed in each video as illustrated in Table 3.

#### 5.2.2. Quantitative Analysis

We used two evaluation metrics for the quantitative analysis: the success rate evaluation and time demand evaluation.

1. Success Rate Evaluation:
  For this, we used the success rate for each frame by following the formula $S\left(f\right)=\frac{\left|r_{t}{⋂}_{}^{}r_{b}\right|}{\left|r_{t}⋃{r_{b}}^{}\right|}$, where *f* shows the frame, S(f) represents the function for the success criterion, $r_{t}$ illustrates the bounding box prediction returned by the tracker, while $r_{b}$ is the ground truth bounding box; finally, we divide the intersection area by the area of the union. To objectively perform the quantitative analysis in each video frame, we count the number of correct tracking frames and divide them by the total number of frames to calculate the success rate.
2. Time Demand Evaluation:
  For this criterion, we used frame rate in terms of FPS or frame per second to measure the time taken by the tracking models for processing the single frame in each video sequence. To equitably measure the time for each frame, we propose T(f)=t, where T(f) is the model’s processing time for the existing frame and *t* is the time it takes for the current frame (f) processing.

#### 5.2.3. Experimental Setup

Hardware: The experiments for all the trackers were conducted on the Nvidia Jetson Xavier NX board. However, the limited memory of the Xavier NX posed a challenge as it does not provide enough space to load and run heavy deep learning models efficiently [92]. To overcome this problem, we created a swap file that allows us to use more memory than the physically installed memory. To do this, we built a memory swap file on the SSD and extended the memory by leveraging the 24GB swap memory with the 8 GB Jetson Xavier NX memory.

Software: The trackers’ implementation has been done in Python using TensorFlow, Keras, OpenCV, and PyTorch platforms, while the detector’s implementation was optimized to run the YOLOv8m model with CUDA on Ubuntu 18.04.

Dataset: We collected the test dataset from real-world environments using both a mobile camera and a ZED camera. Additionally, we downloaded YouTube videos to perform test experiments. The video dataset was designed to capture identities walking in various environments, with each video clip containing different viewpoints, poses, illumination changes, and environment variations in both indoor and outdoor settings, with variations in the number of pedestrians and scenes.

#### 5.2.4. Indoor Environment

**Figure 15:**
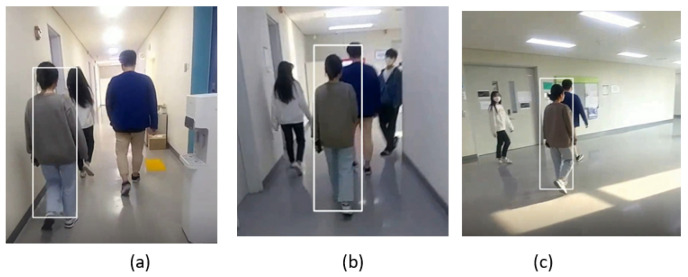
Indoor video_1: Experimental results of the SPT tracker for the qualitative analysis.

A few sample frames of video_1 are shown in Figure 15. It can be seen that the target person is walking in the corridor with two other identities and our SPT tracker is able to track the target from the back side as shown in Figure 15a. When another pedestrian enters the corridor, the SPT tracker does not lose the identity and it tracks the target person as shown in the frame in Figure 15b. We see the light variation frame in Figure 15c when the pedestrians reach a new location. The SPT tracker continues to track the target despite the change in lighting conditions in the scene.

**Figure 16:**
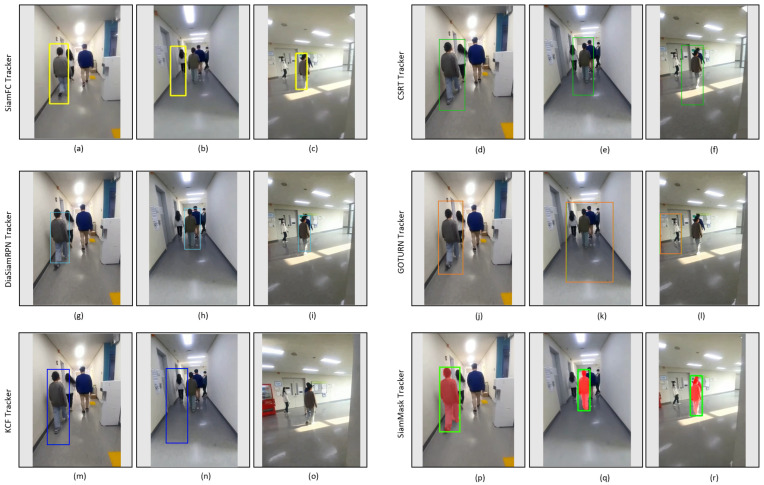
Indoor video_1: Experimental results of SiamFC, CSRT, DiaSiamRPN, GOTURN, KCF, and SiamMask trackers for the qualitative analysis.

Figure 16 shows the tracking results of the tested trackers on the video_1 dataset for comparison with our tracker. This dataset, in some frames, includes partial occlusion and illumination changes, as illustrated in Table 3. In the second frame of the GOTURN tracker, as shown in Figure 16k, it can be seen that it loses its target in the case of partial occlusion and fails to track the target in the following frames shown in Figure 16i, while DiamSiamRPN and SiamMask track the target person in all of the frames. SiamFC and KCF correctly track the objects in the initial frames, as shown in Figure 16a,m. Both trackers fail to track the target after partial occlusion, as shown in Figure 16b,n; however, SiamFC re-detects and tracks the target again despite the illumination variation shown in Figure 16c, while KCF fails as shown in Figure 16o. CSRT properly tracks the target in the few starting frames, yet its performance is poor in the case of illumination variation and partial occlusion, as shown in Figure 16d–f).

The video_1 dataset consists of 444 frames in JPG format. The results of the tracked performance for the quantitative analysis of our tracker compared with SOTA trackers on the video_1 dataset are illustrated in Table 4. It can be seen that the success rate of our tracker is the highest compared to other trackers, followed by SiamMask and DiamSiamRPN trackers. While the performance of SiamFC lies between KCF and CSRT. However, the GOTURN tracker has the lowest success rate.

**Figure 17:**
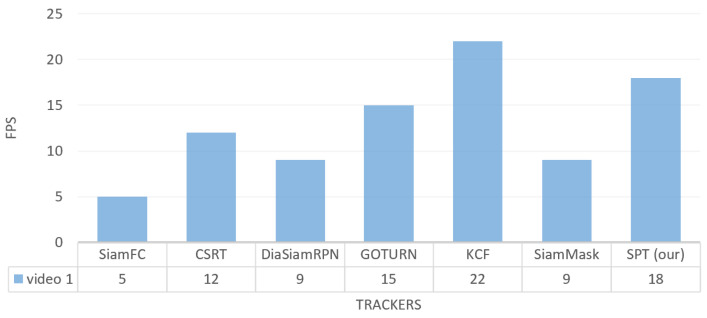
Indoor video_1: Results of the quantitative analysis based on the tracking frame per second (FPS) for the comparison of our SPT tracker with SiamFC, CSRT, DiaSiamRPN, GOTURN, KCF, and SiamMask trackers.

Figure 17 shows the tracking FPS on the video_1 dataset. It can be seen that our tracker has a tracking speed of 18 FPS, which is the highest among all trackers, except KCF, while SiamFC has the lowest tracking speed on the video_1 dataset. CSRT tracks the target with FPS 12, which is faster than SiamMask and DiamSiamRPN, as shown in Figure 17.

**Figure 18:**
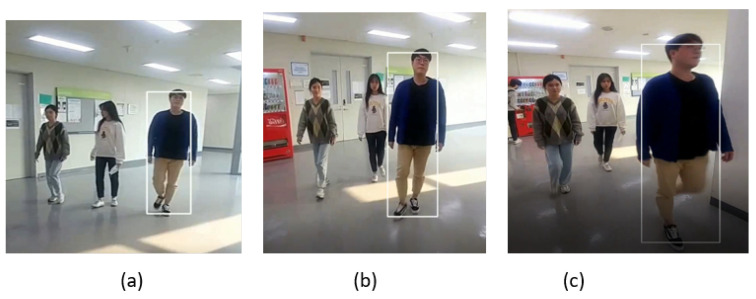
Indoor video_2: Experimental results of the SPT tracker for the qualitative analysis.

The sample frames of the indoor video_2 dataset are shown in Figure 18, where the target pedestrian is tracked from the front view as we can see in the frame in Figure 18a,b. The SPT tracker is also able to track the target from the side pose, as shown in the frame in Figure 18c.

**Figure 19:**
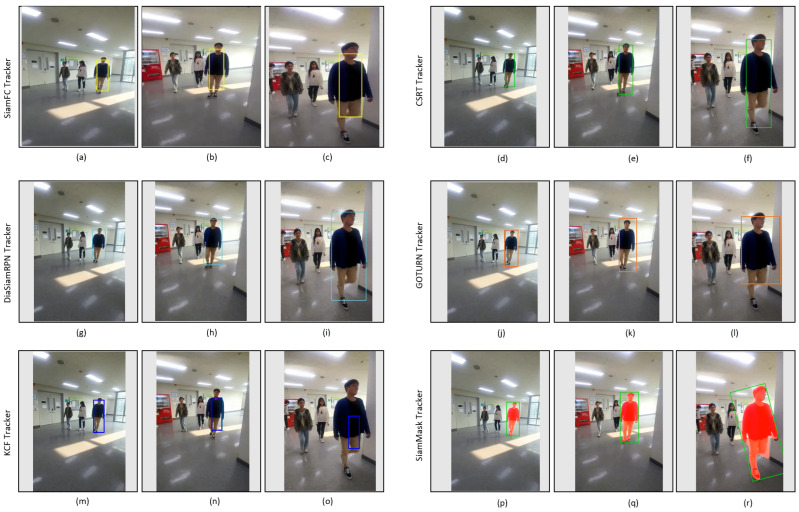
Indoor video_2: Experimental results of SiamFC, CSRT, DiaSiamRPN, GOTURN, KCF, and SiamMask trackers for qualitative analysis.

Figure 19 shows the tracking results of the tested trackers on the video_2 dataset. Its striking points are the presence of appearance changes due to pose variation and illumination changes in the indoor environment, as depicted in Table 3. All the trackers can track the target regardless of light variation, as shown in Figure 19. However, it can be seen that the bounding boxes of SiamFC, GOTURN, and KCF trackers do not scale well to fit the target, as shown in Figure 19c,l,o, while DiamSiamRPN, SiamMask, and CSRT trackers are good despite the pose change (from front pose to side pose) of the target, and the bounding boxes of these trackers also change in scale according to the target size, as shown in Figure 19f,i,r.

The sequence in video_2 has a dataset of 200 frames. The results of our tracker performance compared to the state-of-the-art trackers in the video_2 dataset are shown in Table 5. It can be seen that DiamSiamRPN and SiamFC have the highest performance with an 84% success rate and SiamFC with a 74% success rate. However, the success rate of our tracker is 73%, which is 4%, 3%, 15%, and 2% better than CSRT, GOTURN, KCF, and SiamMask trackers.

**Figure 20:**
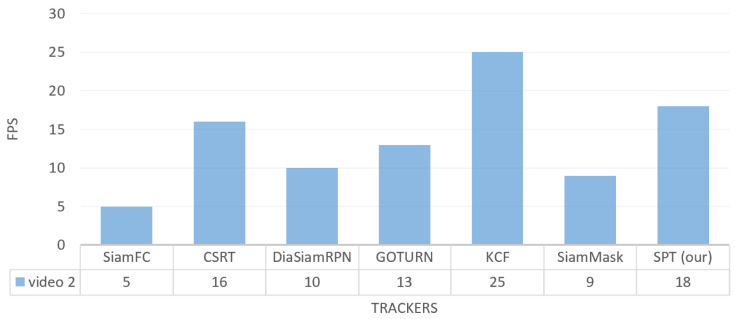
Indoor video_2: Results of quantitative analysis based on the tracking frame per second (FPS) for the comparison of our SPT tracker with SiamFC, CSRT, DiaSiamRPN, GOTURN, KCF, and SiamMask trackers.

Regarding the tracking speed in the video_2 dataset, our tracker shows a tracking speed of 18 FPS, which is faster than SiamFC, SiamMask, DiamSiamRPN, GOTURN, and CSRT, while KCF tracks the target with the highest FPS, as shown in Figure 20.

**Figure 21:**
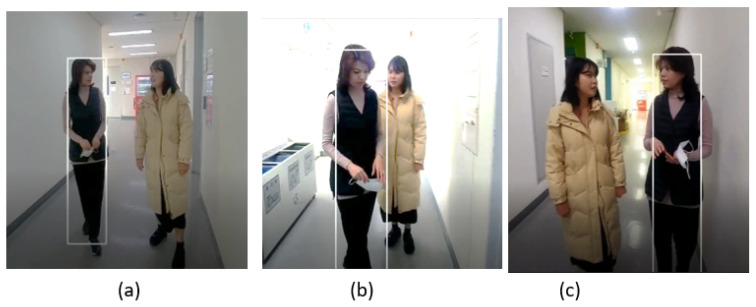
Indoor video_3: Experimental results of the SPT tracker for the qualitative analysis.

In the third indoor video_3 frames dataset, we can see that our SPT tracker successfully tracks the target from the front view as shown in Figure 21. When the target changes position and moves from right to left while walking in the corridor, the SPT tracker does not miss the identity despite the light variation in Figure 21b and the change in the target’s position or location in Figure 21c.

**Figure 22:**
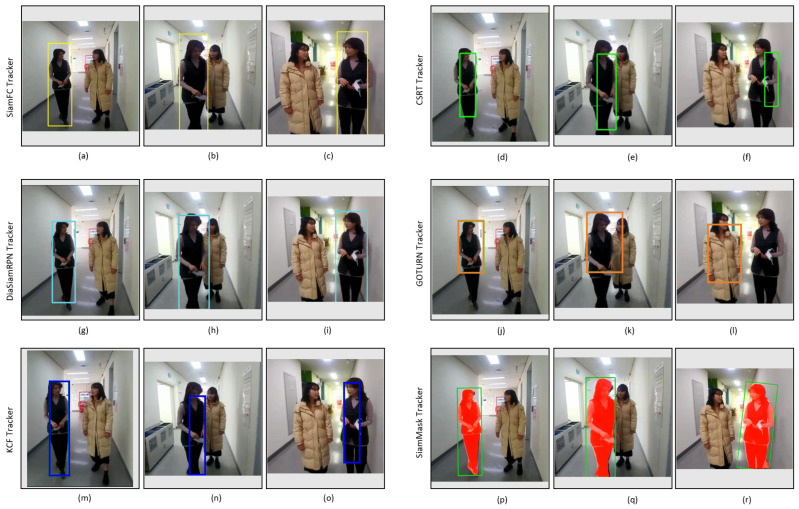
Indoor video_3: Experimental results of SiamFC, CSRT, DiaSiamRPN, GOTURN, KCF, and SiamMask trackers for qualitative analysis.

Figure 22 shows the tracking results of the tested trackers on the video_3 dataset. The substantial factors represented in this dataset include the change in the tracking object’s position, appearance change due to pose variation, and illumination changes in the indoor environment, as listed in Table 3. All the trackers can track the target person, as shown in Figure 22, except the GOTURN tracker, as shown in Figure 22l, where it fails to track when the target changes its position. Moreover, the tracking results of SiamFC, DiamSiamRPN, and SiamMask are better compared to CSRT, KCF, and GOTURN trackers.

The video_3 sequence holds 1495 frames. The quantitative comparison of success rate for our SPT tracker and other trackers on the video_3 dataset is presented in Table 6. The evaluation results show that our SPT tracker, DiamSiamRPN, KCF, and SiamMask trackers achieved a 100% success rate for person tracking in video_3, while the performance of our tracker is 5%, 40%, and 59% higher than SiamFC, CSRT, and GOTURN trackers, respectively.

**Figure 23:**
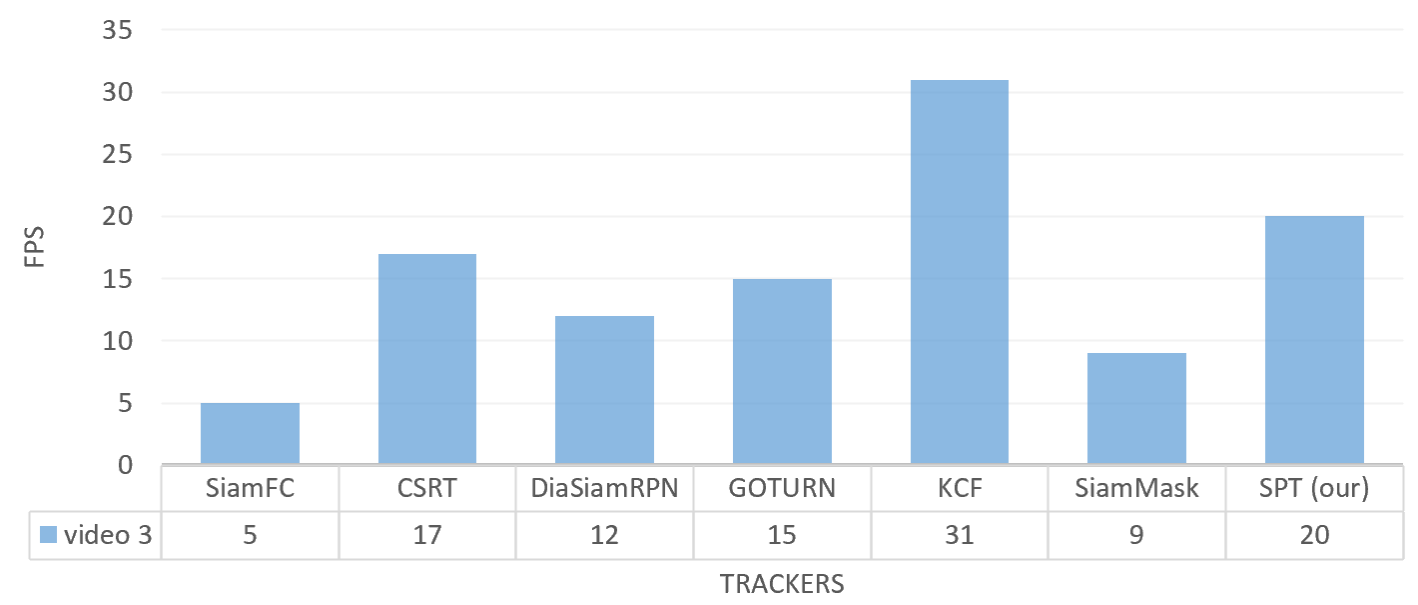
Indoor video_3: Results of the quantitative analysis based on the tracking frame per second (FPS) for the comparison of our SPT tracker with SiamFC, CSRT, DiaSiamRPN, GOTURN, KCF, and SiamMask trackers.

For tracking frames per second on the video_3 dataset, our tracker tracks faster than SiamFC, SiamMask, DiamSiamRPN, GOTURN, and CSRT with FPS 20 while its FPS is lower than the KCF tracker, as shown in Figure 23.

#### 5.2.5. Outdoor Environment

Tracking in the outdoor environment is more challenging compared to the indoor environment.

**Figure 24:**
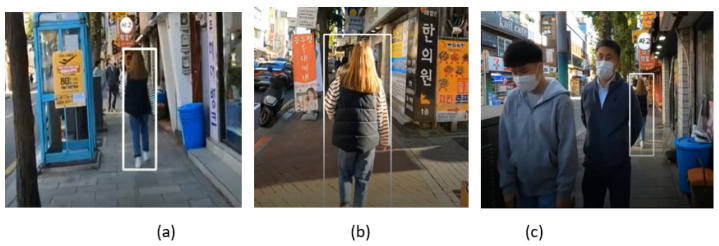
Outdoor video_4: Experimental results of the SPT tracker for the qualitative analysis.

In video_4, we can see that the target person is walking on the footpath in a cluttered outdoor environment while two pedestrians are coming from the front. Our SPT tracker does not lose track of the identity in the case of partial occlusion when the target person passes by other people, as shown in the frame in Figure 24b. It is also able to track the person in varying illumination, as shown in the frame in Figure 24c.

**Figure 25:**
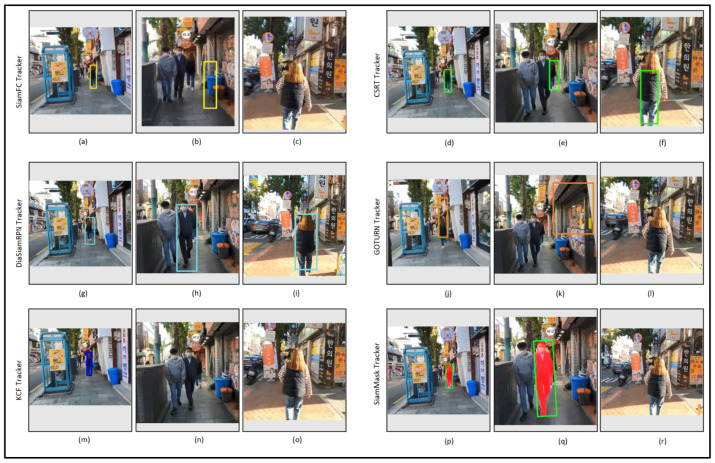
Outdoor video_4: Experimental results of the SiamFC, CSRT, DiaSiamRPN, GOTURN, KCF, and SiamMask trackers for the qualitative analysis.

Figure 25 shows the tracking results of the tested trackers on the video_4 dataset. Two environment factors, known as partial occlusion and illumination variation, were observed in this dataset, as listed in Table 3. All of the trackers successfully track the target person, as shown in Figure 25a,d,g,j,m,p. However, in the next frame, when partial occlusion occurs, as shown in Figure 25b,e,h,k,n,g, all of the trackers fail to find the target as shown in Figure 25c,i,l,o,r, except for the CSRT tracker, which continues to track the target in the cluttered environment, as shown in Figure 25f. However, after some frames, DiamSiamRPN also re-tracks the target and moves the bbox from the car to the target person.

Video_4 contains a sequence of 960 frames. Table 7 shows the success rate evaluation based on the experimental results. It can be seen that our tracker has the highest success rate of 83.1%, which is 1%, 14.2%, 72.1%, and 73.1%, and 65.1% better than CSRT, DiamSiamRPN, GOTURN, SiamFC, and KCF while 37.5% better than KCF and SiamMask trackers, respectively.

**Figure 26:**
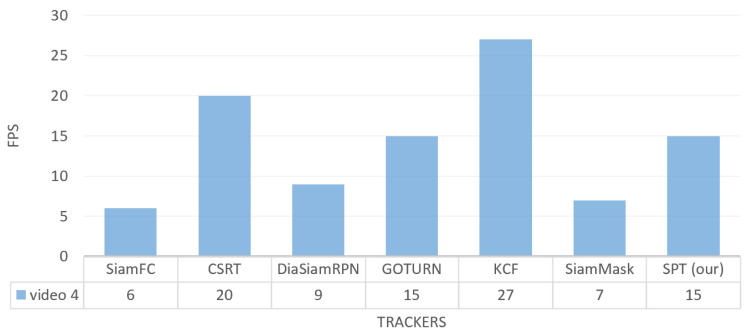
Outdoor video_4: Results of the quantitative analysis based on the tracking frame per second (FPS) for the comparison of our SPT tracker with SiamFC, CSRT, DiaSiamRPN, GOTURN, KCF, and SiamMask trackers.

For frame tracking per second on the video_4 dataset, the speed of our tracker is faster than SiamFC, SiamMask, and DiamSiamRPN with FPS 15, which is the same as the GOTURN tracker, while KCF and CSRT have the highest FPSs (30 and 20, respectively), as shown in Figure 26.

**Figure 27:**
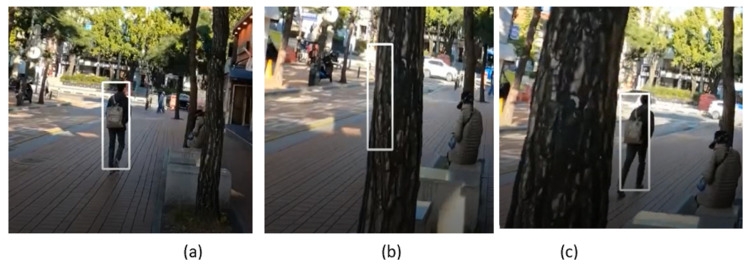
Outdoor video_5: Experimental results of the SPT tracker for the qualitative analysis.

The sample frames from video_5 are shown in Figure 27, where the pedestrian in the street is tracked. After a few video frames, the pedestrian disappears from the scene due to the occlusion of the tree. However, our SPT tracker continues to track the target pedestrian after the full occlusion as shown in frames in Figure 27b,c.

**Figure 28:**
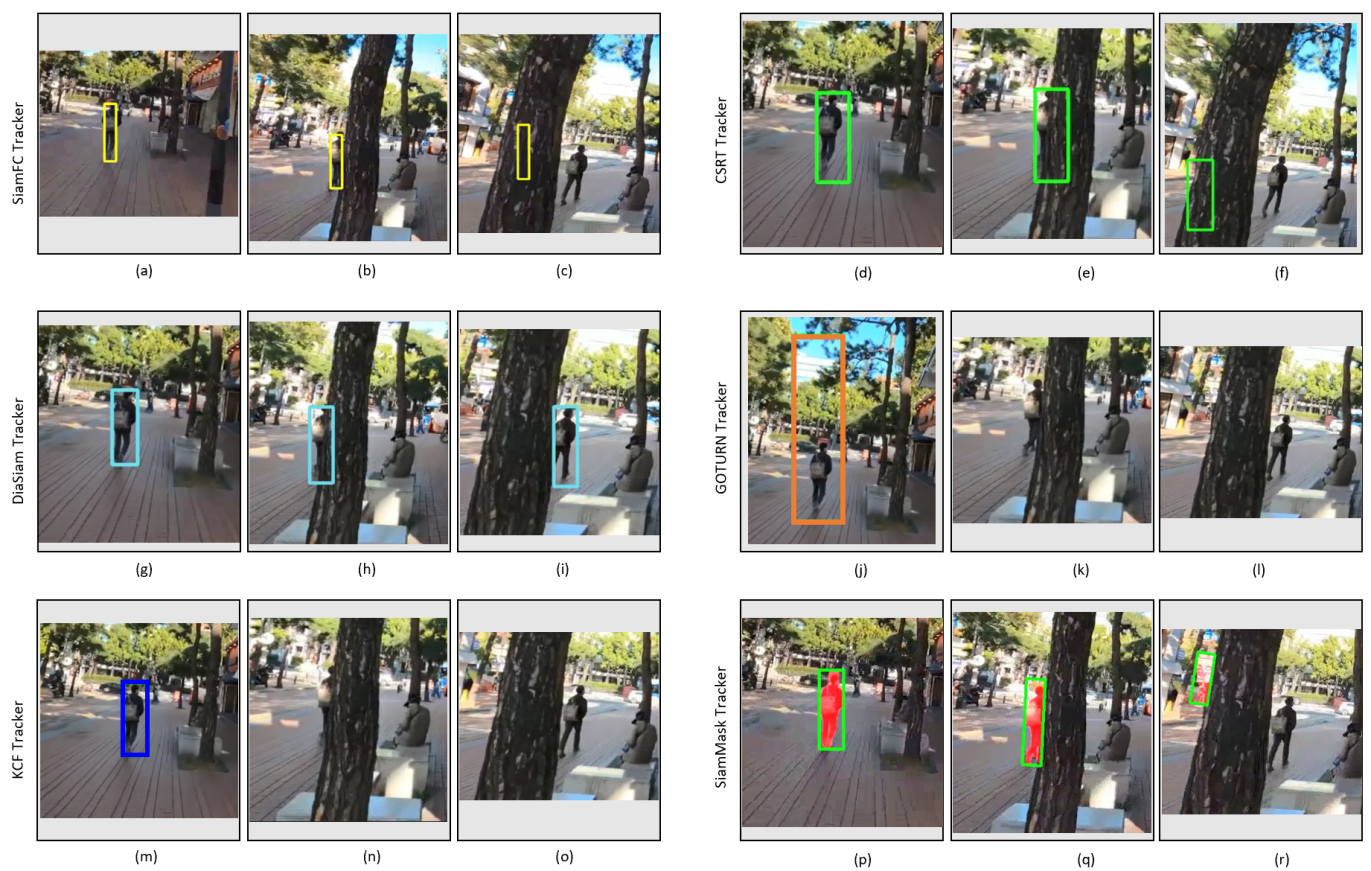
Outdoor video_5: Experimental results of the SiamFC, CSRT, DiaSiamRPN, GOTURN, KCF, and SiamMask trackers for the qualitative analysis.

Figure 28 shows the tracking results of tested trackers on the video_5 dataset. This outdoor dataset covers the environmental factors related to partial occlusion, full occlusion, and illumination variation as listed in Table 3. All the trackers successfully track the target person in the initial frames as shown in Figure 28a,d,g,j,m,p. However, when full occlusion occurs in the next frame as shown in Figure 28b,e,h,k,n,g, all the trackers fail to track the target as shown in Figure 28c,f,l,o,r except DiamSiamRPN tracker which successfully tracks the target after full occlusion as shown in Figure 28i.

This outdoor sequence of video_5 consists of 1276 frames. The tracking results in Table 8 indicate The success rate of our tracker is higher than all other trackers except DiaSiamRPN which has a 6.0% better success rate than our tracker.

**Figure 29:**
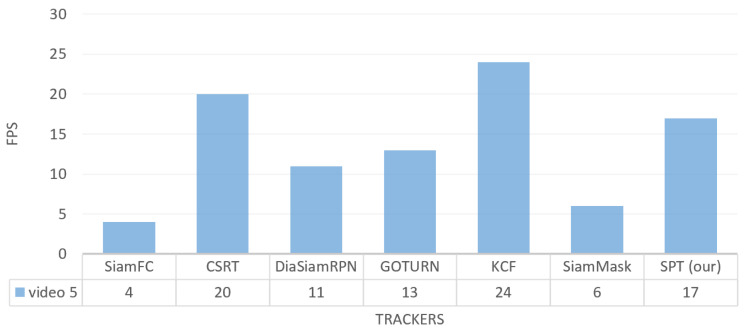
Outdoor video_5: Results of the quantitative analysis based on tracking frame per second (FPS) for the comparison of our SPT tracker with SiamFC, CSRT, DiaSiamRPN, GOTURN, KCF, and SiamMask trackers.

To track frames per second on the video_5 dataset, our tracker runs faster than SiamFC, SiamMask, DiamSiamRPN, and GOTURN with FPS 17, while its speed is lower than CSRT and KCF trackers, as shown in Figure 29.

**Figure 30:**
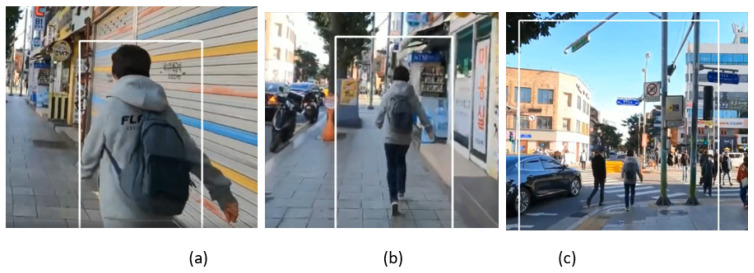
Outdoor video_6: Experimental results of the SPT tracker.

In the video_6 pedestrian tracking scenario, if a person is running, then our SPT tracker drops the tracking identity in the case of the fast-moving target, as shown in Figure 30. In the initial frames, the SPT tracker tracked the pedestrian accurately but it dropped the identity of the target when the pedestrian started to run faster.

**Figure 31:**
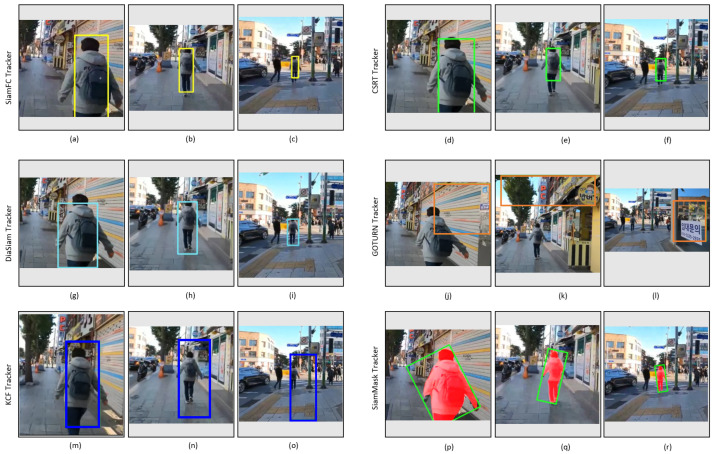
Outdoor video_6: Experimental results of SiamFC, CSRT, DiaSiamRPN, GOTURN, KCF, and SiamMask trackers for the qualitative analysis.

Figure 31 shows the tracking results of the tested trackers on the video_6 dataset. In this dataset, as described in Table 3, the performance of trackers has been analyzed by focusing on the tracking of the fast-moving target. SiamFC, CSRT, DiamSiamRPN, and SiamMask trackers successfully track the target person as shown in Figure 31, while GOTURN fails to track the target. Although KCF successfully tracks the target person in the initial frames, as shown in Figure 31m,n, it also fails to track the target in the case of the fast-moving target, as shown in Figure 31o.

This outdoor dataset of video_6 consists of 955 frames. The quantitative analysis represented in Table 9 shows that the success rate of our tracker is 39.3% better than GOTURN while 0.2% better than KCF. However it is lower than SiamFC, DiaSiamRPN, and SiamMask by 58.7% while 55.7% lower than CSRT in case of fast-moving target.

**Figure 32:**
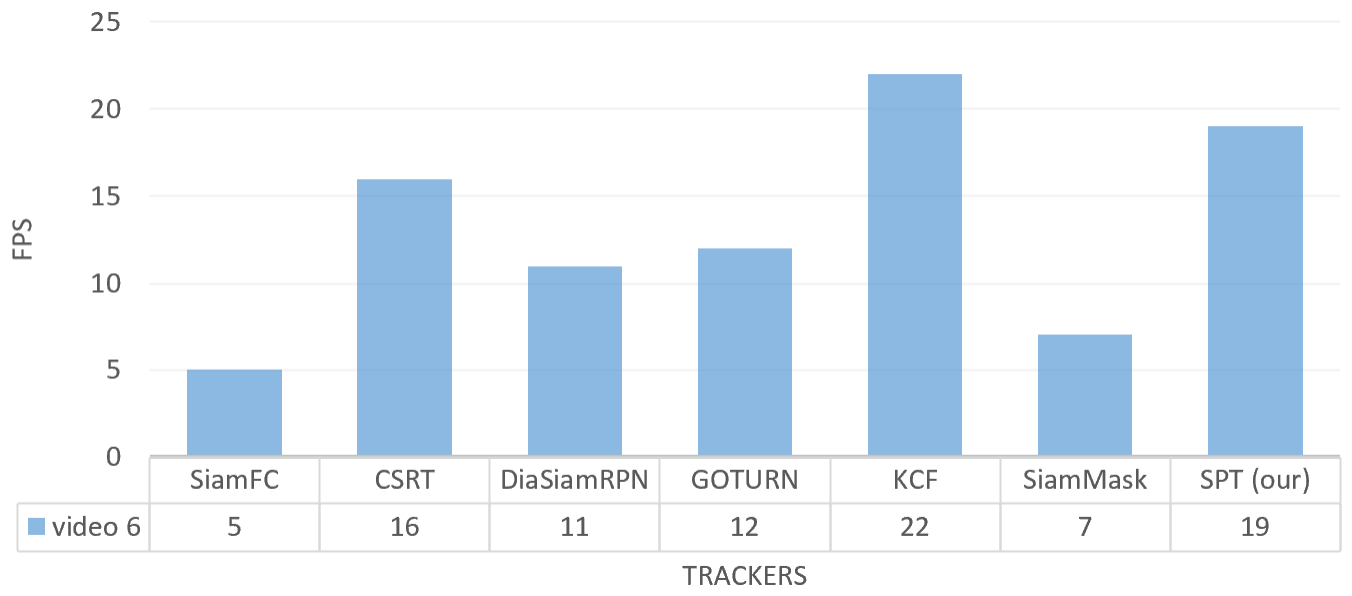
Outdoor video_6: Results of the quantitative analysis based on the tracking frame per second (FPS) for the comparison of our SPT tracker with SiamFC, CSRT, DiaSiamRPN, GOTURN, KCF, and SiamMask trackers.

For frame tracking per second in video_6, our tracker surpasses SiamFC, SiamMask, DiaSiamRPN, GOTURN, and CSRT with FPS 19 while slower than KCF tracker as shown in Figure 32.

To summarize, the above experimental results demonstrate the effectiveness of our SPT tracker for single pedestrian tracking in both indoor and outdoor environments. It can handle appearance variations due to pose changes, illumination variations, changes in position, and slight or short-term partial occlusions and full occlusions. However, its performance affects in case of a fast-moving target or if the target is very far from the camera.

**Figure 33:**
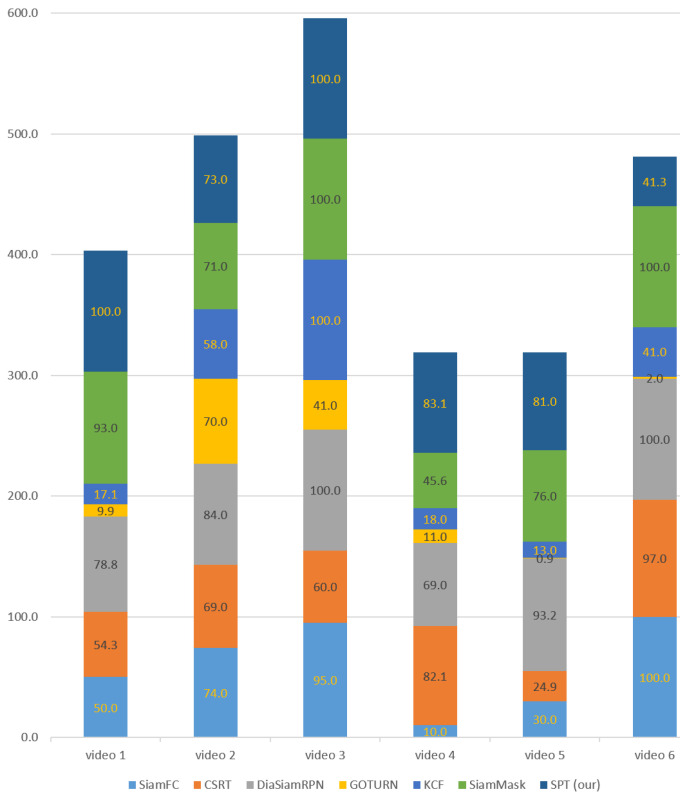
Individual tracking success rate of each video dataset.

- The qualitative analysis, based on the six environmental factors discussed in Table 3, demonstrates that our tracker can handle appearance variation due to pose change, partial occlusion, illumination variation, and change in position. However, its performance is inadequate in the case of fast motion. The qualitative investigation also shows that the overall performance of DiamSiamRPN is better than the other trackers considering all six environmental factors. The performance of the GOTURN tracker is satisfactory in case of appearance variation due to pose change while the performance of KCF is better in case of position change. However, the overall performance of GOTURN and KCF trackers is not satisfactory compared to all others. CSRT’s performance is better than in cases of appearance variant, illumination change, and fast motion. Although SiamMask’s performance is better than SiamFC for a partially occluded target, both trackers fail to track the fully occluded target. The corresponding results based on qualitative comparison are summarized in Table 10.
- The quantitative analysis is conducted by counting the number of correct tracking frames and dividing them by the total number of frames for the success rate evaluation.
  The experimental results in Figure 33 demonstrate that our tracker achieves the highest success rate among other trackers in video_1 (indoor) and video_4 (outdoor) datasets for partial occlusion, illumination variation, pose, and position change factors with success rates of 100% and 83.1%, respectively. In video_2 (indoor), our tracker outperformed KCF, CSRT, GOTURN, and SiamMask trackers with a success rate of 73.0% for appearance variation due to pose change and illumination change. For video_3 (indoor), our tracker achieved a success rate of 100%, which is higher than GOTURN, SiamFC, and CSRT trackers for pose, illumination, and position changes of the target person. However, in video_5 (outdoor), our tracker’s success rate of 81.0% was better than all trackers except DiaSiamRPN. Similarly, in the video_6 dataset, although our tracker’s success rate of 41.3% was higher than GOTURN and KCF trackers, it was still not high enough to effectively track a fast-moving target.

**Figure 34:**
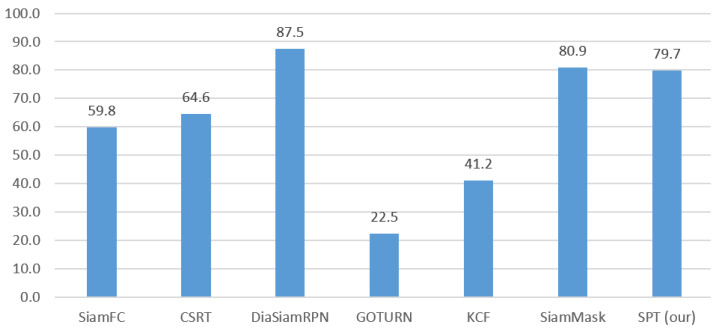
The overall average of the tracking success rate for each tracker on all six video datasets.

To encapsulate, as shown in Figure 34, the overall success rate of all the trackers on six video datasets shows that our tracker lies at the third position with an overall 79.7% success rate which is followed by CSRT, SiamFC, and KCF with 64.6%, 59.8%, and 41.2% success rate while DiamSiamRPN has the highest success rate of 87.5% followed by SiamMask with 80.9% success rate. However, the GOTRUN tracker has the lowest success rate among all which is 22.5%.

**Figure 35:**
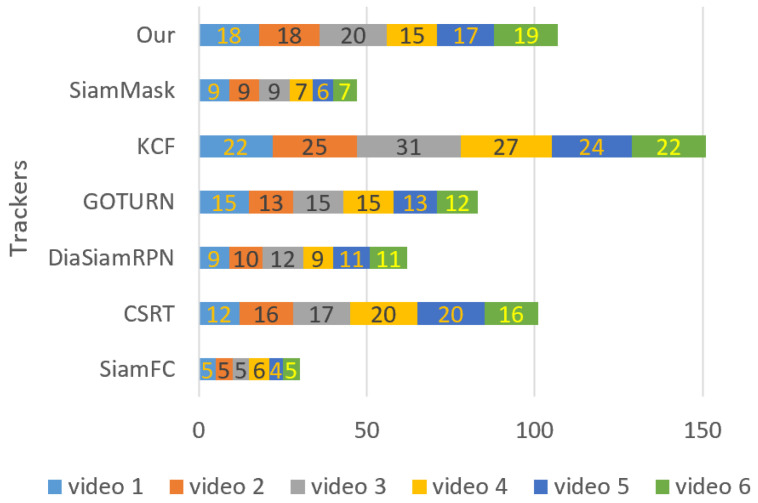
Individual tracking FPS of each video dataset.

The experimental results shown in Figure 35 demonstrate that for frame tracking per second, the overall speed of our tracker is faster than SiamMask, SiamFC, and DiamSiamRPN in all videos, while compared to GOTURN, its FPS is the same as GOTURN FPS in one video, and higher in five video datasets. However, the FPS of our tracker exceeds CSRT in all videos except video_3 and video_4, while KCF is fast among all trackers.

**Figure 36:**
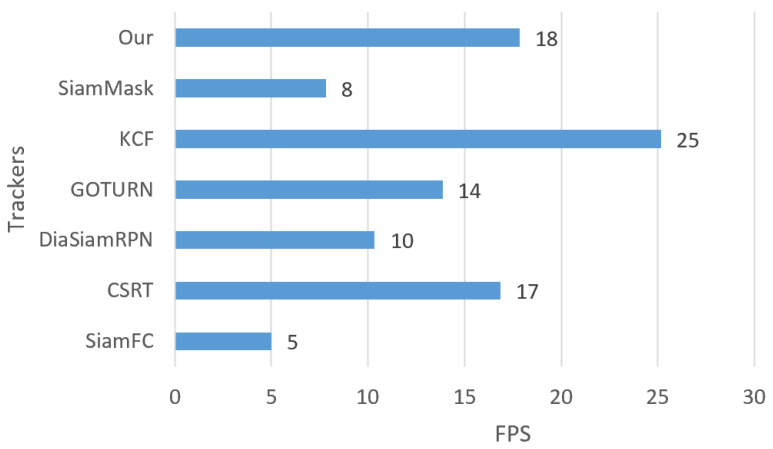
Overall average of the tracking FPS for each tracker on all six video datasets.

To summarize, as shown in Figure 36, regarding the overall frame tracking per second on 6 video datasets, our tracker is in the second position with 18 FPS on average, which is the same as the GOTURN FPS, which is followed by the CSRT, GOTURN, DiamSiamRPN, and SiamMask trackers, with averages FPSs of 17, 14, 10, and 8, respectively. However, KCF has the highest tracking speed with an average of 34 FPS while SiamFC is the slowest among all with 5 FPS on average.

## 6. Conclusions

In this paper, we proposed a single pedestrian tracker (SPT) by combining deep learning and metric learning-based approaches. For this, we used the deep learning-based YOLO model for person detection and introduced metric learning-based DCNet-Siam and MNet-Siam as re-identification models. Both re-identification models were successfully applied to two datasets. The results demonstrated that both models were compact, lightweight, and more accurate compared to eight SOTA person re-identification models. For tracking experiments, we used the DCNet-Siam model for data association through re-identification tasks during the tracking process due to its better performance. In addition to that, we performed qualitative and quantitative analyses of our tracker with six SOTA trackers to demonstrate the effectiveness of our SPT tracker. The overall results confirm the feasibility of the proposed framework for the SPT tracker in indoor and outdoor environments with different illumination changes, appearance variations due to pose changes, changes in the target position, slight or short-term partial occlusion, and full occlusion. However, its limitation is a performance drop in the case of a fast-moving target and the target being too far from the camera. Currently, we are working on part-based human tracking. In the future, we aim to combine a convolutional Siamese network and correlation filter for visual tracking to improve the tracker’s performance.

## Figures and Tables

**Table 1:** Ablation study on the effectiveness of custom components in the base networks of DCNet-Siam and MNet-Siam.

Backbone	#	Component	Rank-1
DCNet	a	Without CLB	0.764
	b	CLB without FMS	0.811
	c	CLB with FMS	0.839
MNet	d	Without CHB	0.756
	e	With CHB	0.792

**Table 3:** Qualitative analysis based on the six environmental factors that were examined in the video sequences.

Video Sequence Name	Environment	Appearance Variation Due to Pose Change	Partial Occlusion	Full Occlusion	Illumination Change	Change in Position	Fast Motion
video_1	Indoor	-	Yes	-	Yes	-	-
video_2		Yes	-	-	Yes	-	-
video_3		Yes	-	-	Yes	Yes	-
video_4	Outdoor	-	Yes	-	Yes	-	-
video_5		-	Yes	Yes	Yes	-	-
video_6		-	-	-	-	-	Yes

**Table 4:** Indoor video_1: Results of the quantitative analysis based on the success rate evaluation for the comparison of our SPT tracker with SiamFC, CSRT, DiaSiamRPN, GOTURN, KCF, and SiamMask trackers.

Trackers	Environment	Total Frames	Frames with Successful Tracking	Frames with Unsuccessful Tracking	Success Rate
SiamFC	Indoor	444	222	222	50.0%
CSRT		444	241	199	54.3%
DiamSiamRPN		444	350	94	78.8%
GOTURN		444	44	400	9.9%
KCF		444	76	368	17.1%
SiamMask		444	413	31	93.0%
SPT (our)		444	444	0	100.0%

**Table 5:** Indoor video_2: Results of the quantitative analysis based on the success rate evaluation for the comparison of our SPT tracker with SiamFC, CSRT, DiaSiamRPN, GOTURN, KCF, and SiamMask trackers.

Trackers	Environment	Total Frames	Frames with Successful Tracking	Frames with Unsuccessful Tracking	Success Rate
SiamFC	Indoor	200	138	62	69.0%
CSRT		200	168	32	84.0%
DiamSiamRPN		200	140	60	70.0%
GOTURN		200	116	84	58.0%
KCF		200	148	52	74.0%
SiamMask		200	142	58	71.0%
SPT (our)		200	146	54	73.0%

**Table 6:** Indoor video_3: Results of the quantitative analysis based on the success rate evaluation for the comparison of our SPT tracker with SiamFC, CSRT, DiaSiamRPN, GOTURN, KCF, and SiamMask trackers.

Trackers	Environment	Total Frames	Frames with Successful Tracking	Frames with Unsuccessful Tracking	Success Rate
SiamFC	Indoor	1495	1420	75	95.0%
CSRT		1495	897	598	60.0%
DiamSiamRPN		1495	1495	0	100.0%
GOTURN		1495	623	882	41.0%
KCF		1495	1495	0	100.0%
SiamMask		1495	1495	0	100.0%
SPT (our)		1495	1495	0	100.0%

**Table 7:** Outdoor video_4: Results of the quantitative analysis based on the success rate evaluation for the comparison of our SPT tracker with SiamFC, CSRT, DiaSiamRPN, GOTURN, KCF, and SiamMask trackers.

Trackers	Environment	Total Frames	Frames with Successful Tracking	Frames with Unsuccessful Tracking	Success Rate
SiamFC	Outdoor	960	96	864	10.0%
CSRT		960	788	172	82.1%
DiamSiamRPN		960	662	298	69.0%
GOTURN		960	106	854	11.0%
KCF		960	173	787	18.0%
SiamMask		960	438	522	45.6%
SPT (our)		768	162	95	83.1%

**Table 8:** Outdoor video_5: Results of the quantitative analysis based on the success rate evaluation for the comparison of our SPT tracker with SiamFC, CSRT, DiaSiamRPN, GOTURN, KCF, and SiamMask trackers.

Trackers	Environment	Total Frames	Frames with Successful Tracking	Frames with Unsuccessful Tracking	Success Rate
SiamFC	Outdoor	1276	383	893	30.0%
CSRT		1276	318	958	24.9%
DiamSiamRPN		1276	1189	87	93.2%
GOTURN		1276	12	1264	0.9%
KCF		1276	166	1110	13.0%
SiamMask		1276	970	306	76.0%
SPT (our)		1276	1033	243	81.0%

**Table 9:** Outdoor video_6: Results of the quantitative analysis based on the success rate evaluation for the comparison of our SPT tracker with SiamFC, CSRT, DiaSiamRPN, GOTURN, KCF, and SiamMask trackers.

Trackers	Environment	Total Frames	Frames with Successful Tracking	Frames with Unsuccessful Tracking	Success Rate
SiamFC	Outdoor	955	955	0	100.0%
CSRT		955	926	29	97.0%
DiamSiamRPN		955	955	0	100.0%
GOTURN		955	19	936	2.0%
KCF		955	392	563	41.0%
SiamMask		955	955	0	100.0%
SPT (our)		955	394	561	41.3%

**Table 10:** Overall performance of each tracker based on the qualitative analysis considering six major factors.

	Appearance Variation Due to Pose Change	Partial Occlusion	Full Occlusion	Illumination Change	Change in Position	Fast Motion
SiamFC	Yes	Poor	Poor	Yes	Yes	Yes
CSRT	Yes	Poor	Poor	Yes	Poor	Yes
DiamSiamRPN	Yes	Yes	Yes	Yes	Yes	Yes
GOTURN	Yes	Poor	Poor	Poor	Poor	Poor
KCF	Poor	Poor	Poor	Poor	Yes	Poor
SiamMask	Yes	Yes	Poor	Yes	Yes	Yes
SPT (our)	Yes	Yes	Yes	Yes	Yes	Poor

## Data Availability

Training and the testing dataset is publically available and open source.

## Abbreviations

The following abbreviations are used in this manuscript:

SPT	Single Pedestrian Tracker
DL	Deep Learning
TL	Transfer Learning
BBox	Bounding box
VOT	Visual Object Tracking
CNN	Convolutional Neural Network
DCNet-Siam	Convolutional Neural Network Siamese
MNet-Siam	Mobile Net Siamese
CUHK	Chinese University of Hong Kong
SOTA	State of the art
LSTM	Long Short-Term Memory
CML	Classical Metric Learning
DML	Deep Metric Learning
SOT	Single Object Tracking
